# TIA-1 promotes FUNDC1-mediated mitophagy to protect against stress-induced cellular senescence

**DOI:** 10.1038/s12276-026-01752-w

**Published:** 2026-06-05

**Authors:** Seongho Cha, Myeongwoo Jung, Hyosun Tak, Seungyeon Ryu, Sukyoung Han, Dongwoo Chae, Jiyoon Kim, Seung Min Jeong, Wook Kim, Eun Kyung Lee

**Affiliations:** 1 Department of Biochemistry, College of Medicine, The Catholic University of Korea, Seoul, South Korea; 2 Department of Medical Science, Graduate School, The Catholic University of Korea, Seoul, South Korea; 3 INSERM U1052, CNRS UMR-5286, Cancer Research Center of Lyon (CRCL), Lyon, France; 4 Department of Pharmacology, Yonsei University College of Medicine, Seoul, South Korea; 5 Department of Pharmacology, College of Medicine, The Catholic University of Korea, Seoul, South Korea; 6 Institute for Aging and Metabolic Diseases, College of Medicine, The Catholic University of Korea, Seoul, South Korea; 7 Department of Molecular Science and Technology, Ajou University, Suwon, South Korea

**Keywords:** Senescence, RNA quality control, Mitochondria

## Abstract

Mitochondrial dysfunction, characterized by reduced mitophagy, excessive mitochondrial elongation, and elevated reactive oxygen species production, is a hallmark of cellular senescence. However, the molecular mechanisms linking impairment of redox balance to mitophagy suppression during senescence remain poorly understood. In this study, we identified TIA-1, an RNA-binding protein, as a positive regulator of FUNDC1 expression, a key receptor for ubiquitin-independent mitophagy. Sodium butyrate and ultraviolet-B irradiation triggered oxidative stress-associated senescence in HaCaT cells, leading to reduced TIA-1 expression, decreased FUNDC1 levels, impaired mitophagy flux, excessive mitochondrial elongation, and upregulation of senescence markers. Conversely, ectopic expression of TIA-1 restored FUNDC1 levels, enhanced mitophagy, improved mitochondrial function, and reduced senescence marker expression. Ribonucleoprotein immunoprecipitation assays confirmed that TIA-1 directly interacts with *FUNDC1* mRNA, and subsequent analyses indicated that TIA-1 enhances FUNDC1 expression primarily through translational control. Together, these findings establish TIA-1 as a pivotal regulator of mitochondrial homeostasis during cellular stress, acting through FUNDC1 to sustain mitophagy and limit senescence. Targeting TIA-1 may offer new strategies to mitigate mitochondrial dysfunction and restore redox balance in aging and age-related diseases.

## Restoring TIA-1 levels improves mitochondrial function in cellular senescence

Mitochondrial homeostasis is crucial for cellular function, influencing energy production and signaling pathways. Disruptions in this balance can lead to diseases such as cancer and neurodegenerative disorders. This study explores the role of TIA-1, an RNA-binding protein, in regulating mitophagy — the process of clearing damaged mitochondria — during cellular senescence. Researchers found that TIA-1 controls the expression of FUNDC1, a receptor crucial for mitophagy. They demonstrated that reduced TIA-1 levels impair mitophagy, leading to mitochondrial dysfunction and cellular aging. By overexpressing TIA-1, they restored mitophagy and reduced senescence markers, highlighting the protective role of TIA-1. These findings suggest that targeting TIA-1 could offer therapeutic potential for conditions involving mitochondrial dysfunction. Future research should explore the mechanisms controlling TIA-1 expression and its broader implications in age-related diseases."This summary was initially drafted using artificial intelligence, then revised and fact-checked by the author."

## Introduction

Mitochondrial homeostasis has a central role in maintaining cellular function by regulating energy production, metabolic processes, and various signaling pathways^1,2^. Dysregulation of mitochondrial homeostasis leads to mitochondrial dysfunction, which has been implicated in several pathological conditions, including neurodegenerative diseases, cancer, and metabolic disorders^2,3^. Cellular senescence, a state of irreversible cell cycle arrest, is characterized by mitochondrial dysfunction^4^. Features of this dysfunction include impaired mitochondrial dynamics, pronounced mitochondrial elongation, increased production of reactive oxygen species (ROS), and accumulation of damaged mitochondria, all of which contribute to the progression of age-related diseases and tissue degeneration^5,6^. In senescent cells, mitochondria exhibit abnormal elongation and dysfunction^7,8^; however, the precise molecular mechanisms underlying these changes remain unclear. Our previous studies have shown that reducing TIA-1 in senescent cells induces mitochondrial elongation^9^. Building on these findings, we further investigated how the loss of TIA-1 contributes to mitochondrial elongation during cellular senescence.

Mitophagy, the selective clearance of damaged mitochondria via autophagy, is essential for maintaining mitochondrial quality and overall cellular homeostasis^10–12^. Although mitophagy has a critical role in preserving mitochondrial integrity, its activity is notably reduced during cellular senescence^10,13^. This decline contributes to the accumulation of dysfunctional mitochondria, exacerbating mitochondrial elongation and ROS production. Despite its recognized importance, the molecular mechanisms underlying the reduction of mitophagy during senescence remain poorly understood, representing a critical gap in our knowledge.

TIA-1, an RNA-binding protein, is another key player in mitochondrial homeostasis^14,15^. It is known to regulate RNA metabolism, including alternative splicing, stability, and translation, and is involved in the formation of stress granules under various stress conditions^16^. TIA-1 has been shown to have an important role in the regulation of mitochondrial homeostasis^14,17,18^. Mutations in TIA-1 have been associated with diseases such as amyotrophic lateral sclerosis and dementia^19^, highlighting its potential clinical importance. However, the precise role of TIA-1 in mitophagy and its regulatory mechanisms during cellular senescence remain largely unexplored.

In this study, we investigated the molecular mechanisms by which TIA-1 regulates mitochondrial homeostasis and mitophagy during stress-induced cellular senescence. Our results showed that TIA-1 regulates the expression of FUNDC1, a mitochondria-specific receptor of mitophagy^20^. Furthermore, we showed that the reduction of TIA-1 during cellular senescence leads to decreased FUNDC1 expression, which in turn impairs mitophagy. These findings provide new insights into how TIA-1 downregulation contributes to mitochondrial dysfunction in senescent cells. By providing new insights into the molecular regulation of mitophagy and mitochondrial homeostasis, this study has the potential to advance our understanding of cellular senescence and its contribution to age-related and mitochondria-associated diseases. In addition, this work highlights TIA-1 as a potential therapeutic target for alleviating mitochondrial dysfunction in pathological conditions.

## Materials and methods

### Cell culture and transfection

HaCaT (human skin keratinocytes), HEK293T (human embryonic kidney cells), and SH-SY5Y (human neuroblastoma) were cultured in DMEM (Cytiva, Wilmington, DE, USA) supplemented with 10% fetal bovine serum and 1% antibiotics at 37 °C. Plasmids overexpressing FUNDC1 and DRP1 were generated by inserting each open reading frame into pCMV6-AN-His-HA mammalian expression vector using specific primer sets (Supplementary Table 1), whereas pTIA-1 and pMFF were previously generated and validated in our prior work^9^. Transfection of small interfering RNAs (siRNAs) against TIA-1 and FUNDC1 (Genolution Pharmaceuticals, Inc., Seoul, South Korea and Bioneer, Daejeon, South Korea), plasmids, and their appropriate controls was carried out using Lipofectamine^TM^ 2000 (Invitrogen^TM^), according to the manufacturer’s instructions.

To establish the stress-induced senescence model, HaCaT cells were incubated with media containing 1 mM sodium butyrate (NaBu) (Sigma-Aldrich, Burlington, MA, USA) or exposed to ultraviolet (UV)-B (75 mJ/cm^2^) using UV Transilluminator (Core Bio System, Seoul, Korea) during the indicated time.

### RNA analysis

Total RNA was extracted from whole cells using RNAiso Plus (Takara Bio, Inc., Shiga, Japan), and cDNA was synthesized by reverse transcription (RT) with the ReverTra® Ace qPCR RT kit (Toyobo Co., Ltd, Osaka, Japan). Quantitative PCR (qPCR) was conducted with the SensiFAST™ SYBR Hi-ROX kit (Meridian Bioscience, Inc., Cincinnati, OH, USA) and gene-specific primer sets listed in Supplementary Table 1 using the StepOnePlus™ Real-Time PCR System (Applied Biosystems, Waltham, MA, USA). Relative levels of mRNAs were calculated using the ^ΔΔ^CT method, comparing control and each experimental group. *GAPDH* mRNA was used as an internal reference gene for normalization.

Stability of *FUNDC1* mRNA was determined by the Click-iT™ Nascent RNA Capture kit (Invitrogen), according to the manufacturer’s instructions. Briefly, cells were labeled with 0.2 mM ethynyl uridine (EU) at 37 °C and then recovered in EU-free medium for 0, 2, 4, or 8 h, respectively. After extracting total RNA, the EU-labeled RNAs were metabolically labeled with biotin, purified with Dynabeads® MyOne™ Streptavidin T1 magnetic beads, and analyzed by RT–qPCR using the primer sets for *FUNDC1* mRNA.

### Western blot (WB) analysis

Whole-cell lysates were prepared using RIPA buffer containing 1× protease inhibitor cocktail (Roche, Basel, Switzerland). The samples were separated by SDS–PAGE and transferred onto polyvinylidene difluoride membranes. The membranes were incubated overnight at 4 °C with primary antibodies against TIA-1, p21, p16, GAPDH, p-IRF3, ISG15, GFP, Lamin B, TOM40, FUNDC1, BCL2L13, NIX, BNIP3, MFF, HA, DRP1, Streptavidin-horseradish peroxidase, or β-actin (Supplementary Table 2). Following incubation with primary antibodies, the membranes were further incubated with HRP-conjugated secondary antibodies at room temperature for 1 h. Chemiluminescence was detected by applying the Clarity Western ECL Substrate (Bio-Rad, Inc., Hercules, CA, USA) to the membranes, and luminescent signals were acquired using the ChemiDoc Imaging Systems (Bio-Rad, Inc.).

To analyze de novo protein synthesis, newly synthesized proteins were isolated using the Click-IT AHA (L-Azidohomoalanine) kit (Invitrogen), according to the manufacturer’s instructions. In brief, cells were incubated in methionine-free media for 1 h and labeled with 50 μM AHA. The nascent AHA-labeled proteins were then labeled with biotin, isolated using Dynabeads™ M-280 Streptavidin (Invitrogen), and analyzed by WB using a streptavidin-HRP conjugate (Invitrogen) and FUNDC1 antibody.

### Analysis of mitochondrial morphology

Cells were incubated with 100 nM MitoTracker Red CMXRos (Invitrogen) in serum-free medium for 30 min at 37 °C. Mitochondrial morphology was observed by tracing fluorescent signals using the Observer Z1 inverted microscope (Carl Zeiss, Oberkochen, Germany) and analyzed using the Mitochondrial Analyzer plugin for ImageJ/Fiji software (https://github.com/AhsenChaudhry/Mitochondria-Analyzer). Median value in control group was used as an intermediated group^21^. The elongated group refers to cells with increased mitochondrial length compared with the control. Mitochondrial elongation was determined by measuring the ratio of cells that have elongated forms of mitochondria to total cells.

For transmission electron microscopy (TEM) analysis, cells were fixed with 1% osmium tetroxide and embedded in Epon 812. Ultrathin sections were analyzed with a TEM (JEM 1010, Tokyo, Japan). The perimeter of mitochondria in the TEM image of each experimental group was analyzed using ImageJ software^9,14^.

### mt-Keima assay

The modified mt-Keima reporter plasmid was generated by cloning the fragment corresponding to the COX8/COX8/hmKeima-Red sequence from pCHAC-mt-mKeima (Addgene no. 72342) into the pcDNA 3.1. Following transfection with the pcDNA 3.1-mt-Keima plasmid (phmt-Keima), cells were incubated with 20 μM carbonylcyanide 4-(trifluoromethoxy)phenylhydrazone (FCCP) and 5 μM oligomycin (Sigma-Aldrich) for 6 h, and then the mt-Keima signal was observed using the Observer Z1 inverted microscope (Carl Zeiss) to detect acidic (586 nm excitation, “red”) and pH-neutral (440 nm excitation, “green”) signals. Mitophagy events were determined by calculating the ratio of red (mitophagy) to green (mitochondria) fluorescence signals from microscopic images using ImageJ software.

### Immunostaining and fluorescence microscopy

Cells were labeled with 100 nM LysoTracker Red DND-99 (MedChemExpress, Monmouth Junction, NJ, USA) in serum-free medium for 30 min at 37 °C, followed by fixation with 4% formaldehyde at room temperature. After permeabilization with Triton X-100 solution, cells were sequentially incubated with blocking solution and primary antibody against NDUFV2, a subunit of mitochondrial complex I located in the inner membrane, followed by incubation with Alexa Fluor® 488-conjugated secondary antibodies (Supplementary Table 2). Nuclei were stained with 4′,6-diamidino-2-phenylindole (Invitrogen). Fluorescence images were acquired using an Observer Z1 inverted fluorescence microscope (Carl Zeiss) with consistent imaging parameters across all conditions.

The number of lysosomes–mitochondria overlay events was manually quantified by counting discrete yellow puncta, indicative of colocalization, within individual cells as described in ref. ^22^. At least 200 cells selected randomly per condition were analyzed.

### Analysis of mitochondrial function

Mitochondrial membrane potential (Δ*ψ*_m_) and ATP generation were measured using the JC-1 assay kit (Abcam, Cambridge, UK) and the Mitochondrial ToxGlo™ system (Promega, Madison, WI, USA), respectively, according to the manufacturers’ protocols. JC-1 fluorescence was detected at 535 nm (excitation) and 590 nm (emission), whereas ToxGlo signals were recorded at 485 nm/530 nm using a Synergy H1 microplate reader (BioTek, Santa Clara, CA, USA).

Mitochondrial ROS levels were assessed by incubating cells with 2.5 μM MitoSOX™ Red (Invitrogen) in Hanks’ Balanced Salt Solution for 15 min. Fluorescence images were acquired using the Observer Z1 inverted fluorescence microscope (Carl Zeiss), and signal quantification was performed using ImageJ software.

### Analysis of cytosolic mitochondrial DNA release

Cytosolic mitochondrial DNA (mtDNA) was analyzed as described in refs. ^23,24^. In brief, the cytosolic fraction was isolated by differential centrifugation using a lysis buffer containing 50 mM HEPES (pH 7.4), 150 mM NaCl, 18 μg/ml digitonin, and 1× protease inhibitor cocktail. Genomic DNA was extracted from the cytosolic fraction using the AccuPrep® Genomic DNA Extraction Kit (Bioneer), according to the manufacturer’s instructions. Cytosolic mtDNA levels were quantified by qPCR using gene-specific primers for *MT-ND1* and *MT-D-loop* (Supplementary Table 1) on a StepOnePlus Real-Time PCR System (Applied Biosystems™). For normalization, cytosolic mtDNA (relative level) was calculated by normalizing the mtDNA abundance in the cytosolic fraction to the total cellular mtDNA content measured from genomic DNA isolated from whole-cell lysates, with *KCNJ10* used as the nuclear reference gene.

### Senescence-associated β-galactosidase assay

Cells were fixed in 4% formaldehyde and incubated in a staining solution containing 40 mM citric acid/sodium phosphate (pH 6.0), 150 mM sodium chloride, 2 mM magnesium chloride, 5 mM potassium ferricyanide, 5 mM potassium ferrocyanide, and 1 mg/mL X-gal (BEAMS Biotechnology, Seongnam, South Korea) at 37 °C for 16 h in the dark^9^. After fixation in 20% glycerol, β-galactosidase levels were observed using an IX70w microscope (Olympus Corp., Tokyo, Japan). The number of β-gal-positive cells was counted from at least 150 cells per experiment, across three independent experiments.

### Ribonucleoprotein immunoprecipitation (RNP IP)

RNP complexes were immunoprecipitated from cell lysates using Protein A beads (Invitrogen) coated with anti-TIA-1 or normal rabbit IgG antibody (Santa Cruz Biotechnology, Inc., Dallas, TX, USA). The immunoprecipitated RNP complexes were then sequentially treated with DNase I and proteinase K. RNAs isolated from the complexes were analyzed by RT–qPCR using gene-specific primer sets listed in Supplementary Table 1.

### Biotin pull-down assay

T7 promoter-incorporated DNA fragments corresponding to the 3′ untranslated region (3′UTR) of *FUNDC1* mRNA (NM_173794.4) were amplified by PCR using specific primers (Supplementary Table 1). Biotinylated RNA probes were synthesized by in vitro transcription using the MaxiScript T7 kit (Ambion, Waltham, MA, USA) and biotin-CTP (Enzo Life Sciences, Farmingdale, NY, USA). Cell lysates were incubated with purified biotinylated RNA probes for 30 min at room temperature. Proteins bound to biotinylated RNA probes were isolated using streptavidin magnetic beads (Invitrogen) and further analyzed by WB using TIA-1 antibody^9^.

### EGFP reporter analysis

The EGFP reporter containing the 3′UTR of *FUNDC1* mRNA (pEGFP-FUNDC1 3U) was generated by inserting the 3′UTR fragment of *FUNDC1* mRNA (NM_173794.4, 496–1,054 nt, 559 bp) into pEGFP-C1 plasmid (BD Bioscience, Franklin Lakes, NJ, USA). After transfection with siRNA (siCtrl or siTIA-1) or plasmid (pCtrl or pTIA-1), the cells were sequentially transfected with reporter plasmids (control C1 or EGFP-FUNDC1 3U) for 24 h. The relative expression of the EGFP reporter was assessed by WB analysis.

### Statistical analysis

Data are presented as the mean ± SEM from three independent experiments. Statistical significance was assessed using Student’s *t-*test for comparison of two groups and one-way analysis of variance followed by Dunnett’s post hoc test for multiple comparisons, as appropriate (n.s., not significant with *P* > 0.05; *P < 0.05; **P < 0.01; ***P < 0.001; ****P < 0.0001).

## Results

### Stress-induced senescence is associated with mitochondrial hyperfusion and reduced mitophagy in HaCaT cells

**Fig. 1:**
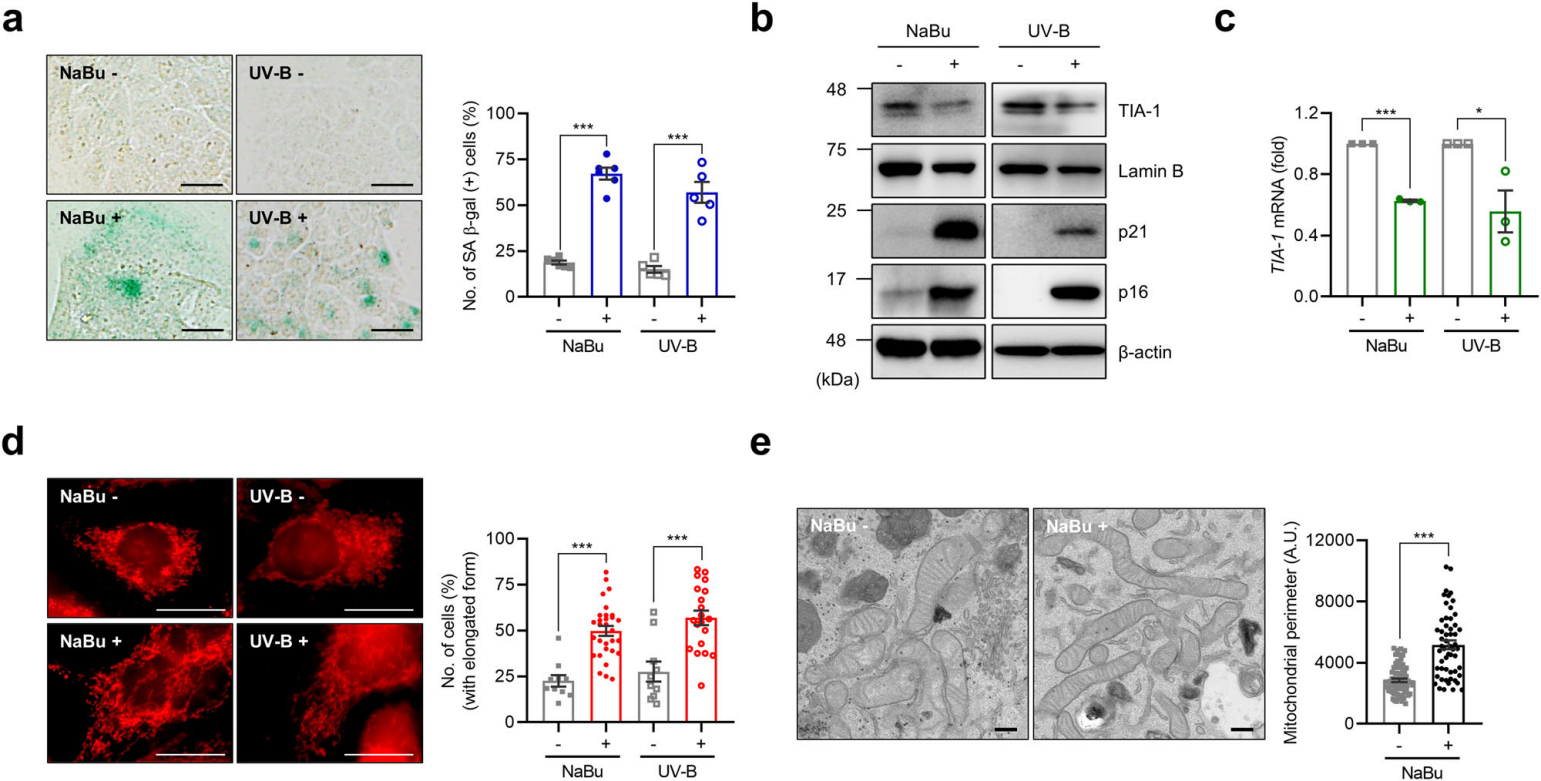
Mitochondrial hyperfusion is induced by stress in HaCaT cells. HaCaT cells were treated with 1 mM sodium butyrate (NaBu) or exposed to ultraviolet (UV)-B irradiation (75 mJ/cm²), followed by incubation for 72 h. **a**, Senescence-associated β-galactosidase (SA β-gal) activity was assessed by X-gal staining (pH 6.0) and the number of SA β-gal-positive cells was quantified. **b**,**c**, TIA-1 expression was analyzed by western blotting and RT–quantitative PCR. β-Actin is a loading control for western blotting, and *GAPDH* mRNA was used for RT–quantitative PCR normalization. Mitochondrial morphology was analyzed using MitoTracker staining (part **d**) and transmission electron microscopy (part **e**). The number of cells with elongated mitochondria and the mitochondrial perimeter were determined using ImageJ software. Representative images are shown and data represent mean ± SEM from three independent experiments. A.U., arbitrary unit. Scale bars: 20 μm (parts **a** and **d**) and 0.5 μm (part **e**). *P < 0.05; ***P < 0.001.

To establish a model of stress-induced senescence in human keratinocyte HaCaT cells, we treated cells with NaBu or UV-B irradiation. Both stressors significantly increased senescence-associated β-galactosidase (SA β-gal) activity, as demonstrated by enhanced SA β-gal staining in treated cells (Fig. 1a). WB analysis revealed that NaBu and UV-B exposure upregulated the expression of canonical senescence markers p16 and p21, while reducing lamin B levels (Fig. 1b). Notably, both treatments also led to a marked decrease in the expression of TIA-1 (Fig. 1b), as observed in our previous study^9^. Consistently, RT–qPCR analysis confirmed that *TIA-1* mRNA levels were significantly reduced under both stress conditions (Fig. 1c). Next, we examined mitochondrial morphology using MitoTracker staining. Both NaBu and UV-B treatments significantly increased the proportion of cells exhibiting elongated mitochondria, indicating stress-induced mitochondrial hyperfusion (Fig. 1d). TEM analysis further showed that NaBu treatment increased mitochondrial perimeter compared with controls (Fig. 1e).

**Fig. 2:**
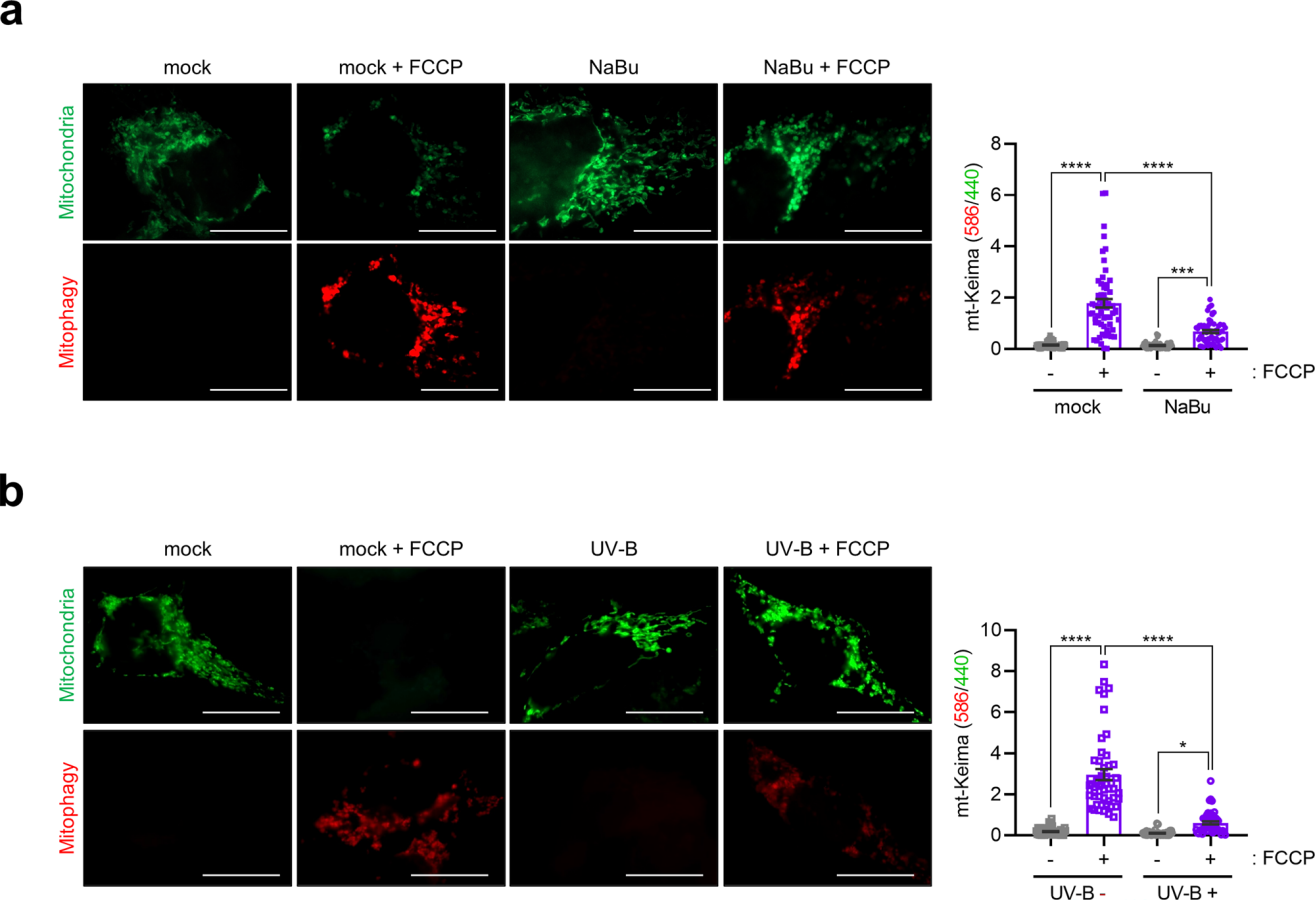
Mitophagy is reduced in stress-induced senescence. After transfection with phmt-Keima, HaCaT cells were incubated with sodium butyrate (NaBu) (part **a**) or exposed to ultraviolet (UV)-B irradiation (75 mJ/cm²) (part **b**) for 72 h. Mitophagy activity was assessed by monitoring the red fluorescence signals of the mt-Keima reporter following treatment with FCCP (20 μM) and oligomycin (5 μM) for 6 h. The fluorescence ratio (red/green) was determined using ImageJ software, as described in the Materials and methods section. Representative images are shown and data represent mean ± SEM from three independent experiments. Scale bar, 20 μm. *P < 0.05; ***P < 0.001; ****P < 0.0001.

The enhanced mitochondrial elongation in senescent cells could result from the altered expression of proteins that regulate mitochondrial dynamics^6,23^; however, it could also be due to a decrease in mitophagy activity, a form of selective autophagy leading to mitochondrial degradation^25,26^. To investigate the alteration in mitophagy in cellular senescence, we assessed mitophagy activity in these stress conditions using the mt-Keima reporter assay^27,28^. HaCaT cells transfected with mt-Keima were treated with NaBu or UV-B and then challenged with FCCP to induce mitophagic flux. Both NaBu and UV-B treatment significantly reduced the FCCP-induced mt-Keima red-to-green fluorescence ratio, indicating impaired mitophagy activity under stress (Fig. 2a,b). Together, these findings demonstrate that NaBu and UV-B-induced stress promote cellular senescence in HaCaT cells, which is associated with decreased TIA-1 expression, mitochondrial hyperfusion, and reduced mitophagic clearance.

### TIA-1 knockdown exacerbates stress-induced senescence and suppresses mitophagy

**Fig. 3:**
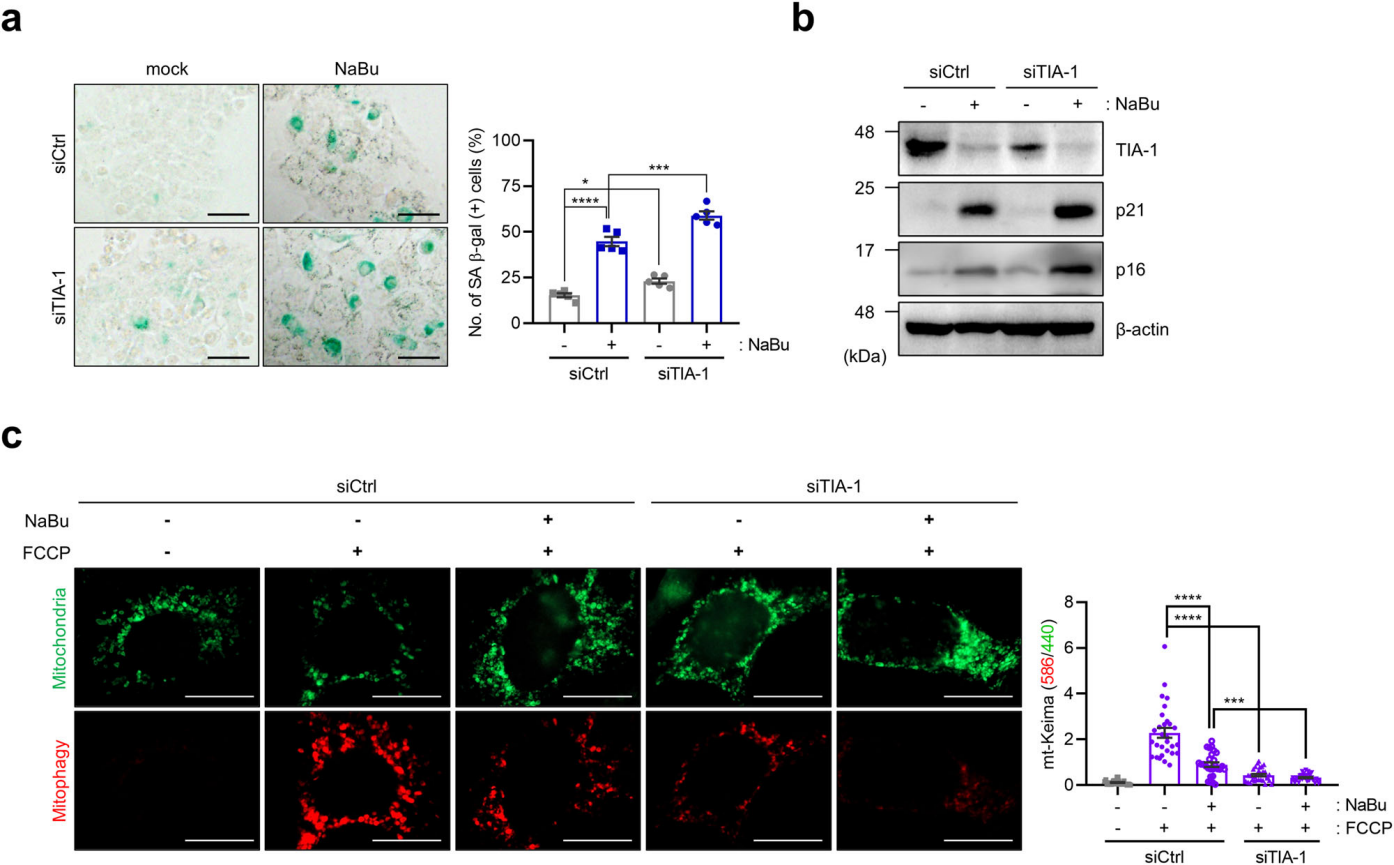
TIA-1 knockdown promotes cellular senescence and reduces mitophagy in response to NaBu treatment. Following TIA-1 knockdown, HaCaT cells were treated with 1 mM sodium butyrate (NaBu) for 48 h. **a** Senescence-associated β-galactosidase (SA β-gal) activity was assessed by X-gal staining (pH 6.0) and the number of SA β-gal-positive cells was quantified. **b** Protein expression was analyzed by western blotting. β-Actin was used as the loading control. **c** Mitophagy activity was assessed by monitoring the red fluorescence signals of the mt-Keima reporter following treatment with FCCP (20 μM), and the fluorescence ratio (red/green) was determined using ImageJ software. Representative images are shown and data represent mean ± SEM from three independent experiments. Scale bar, 20 μm. *P < 0.05; **P < 0.01; ***P < 0.001; ****P < 0.0001.

To investigate the role of TIA-1 in regulating mitophagy under stress conditions, we first assessed mt-Keima reporter activity after TIA-1 knockdown. TIA-1 downregulation significantly reduced FCCP-induced mt-Keima red fluorescence signal in HaCaT, HEK293T, and SH-SY5Y cells (Supplementary Fig. 1), indicating that TIA-1 downregulation broadly impairs mitophagic flux. Next, we examined how TIA-1 knockdown influenced stress-induced senescence and mitophagy in the NaBu treatment model. In HaCaT cells, TIA-1 knockdown led to a greater increase in SA β-gal-positive staining (Fig. 3a) and an elevation of senescence markers p21 and p16 (Fig. 3b) after NaBu treatment compared with control. Mitophagy activity, measured by the mt-Keima assay, was even more decreased by TIA-1 knockdown in NaBu-treated cells, as reflected by a further reduced red-to-green fluorescence ratio (Fig. 3c). Similar enhancement of NaBu-induced mitophagy suppression was observed in HEK293T cells following TIA-1 downregulation (Supplementary Fig. 2). These findings suggest that the loss of TIA-1 level exacerbates stress-induced senescence and further impairs mitophagic clearance in HaCaT cells.

### TIA-1 overexpression mitigates stress-induced senescence and restores mitophagy activity

**Fig. 4:**
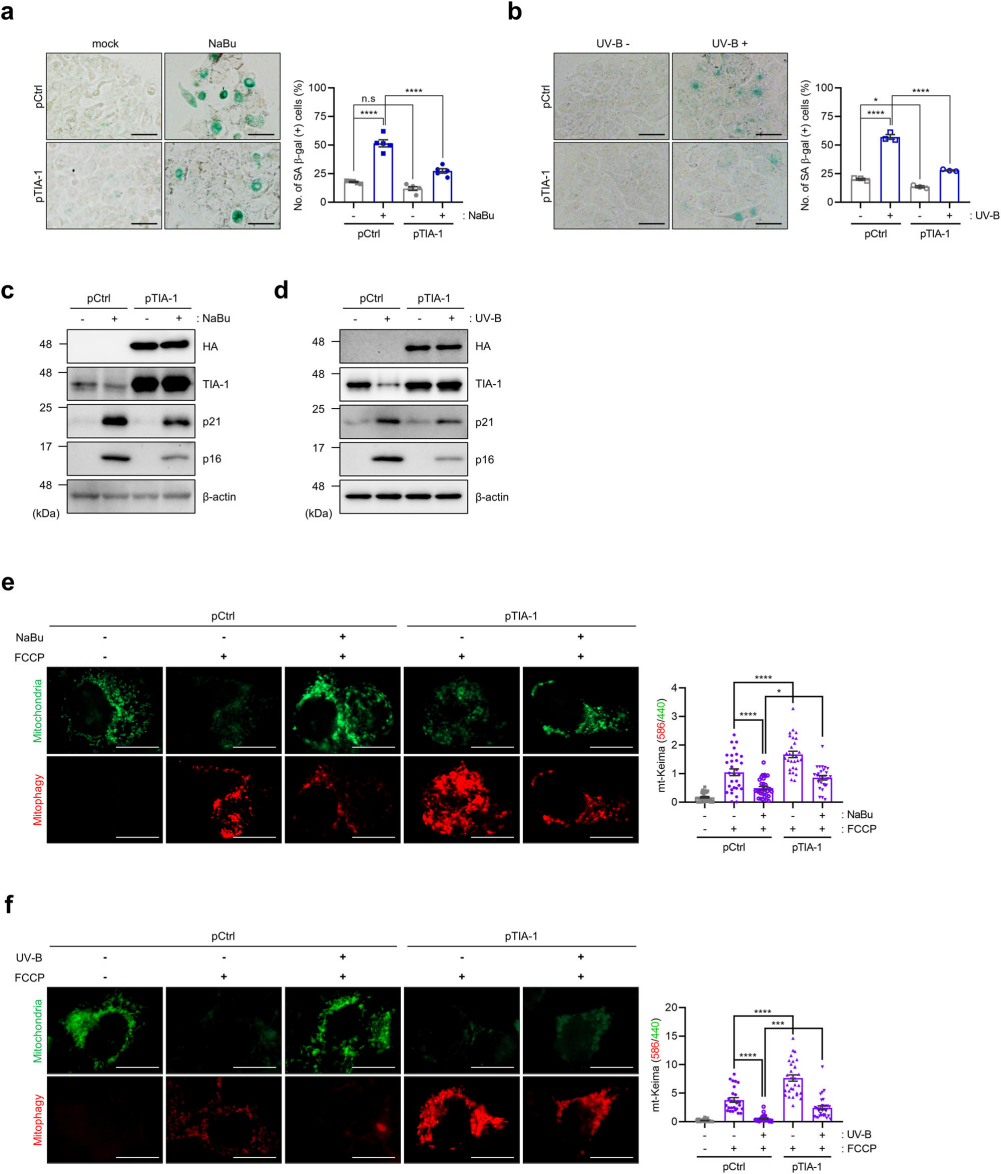
TIA-1 overexpression attenuates mitophagy reduction during stress-induced senescence. Following ectopic expression of pTIA-1, HaCaT cells were treated with 1 mM sodium butyrate (NaBu) or exposed to ultraviolet (UV)-B (75 mJ/cm^2^) and incubated for 48 h. Senescence-associated β-galactosidase (SA β-gal) activity was assessed by X-gal staining (pH 6.0) and the number of SA β-gal-positive cells was quantified in NaBu-treated (part **a**) and UV-B-treated (part **b**) groups. Protein expression was analyzed by western blotting in NaBu-treated (part **c**) and UV-B-treated (part **d**) groups. β-Actin was used as the loading control. Mitophagy activity was assessed by monitoring the red fluorescence signals of the mt-Keima reporter following treatment with FCCP (20 μM) and the fluorescence ratio (red/green) was determined in NaBu-treated (part **e**) and UV-B-treated (part **f**) groups using ImageJ software. Representative images are shown and data represent mean ± SEM from three independent experiments. Scale bar, 20 0μm. n.s., not significant; *P* > 0.05; *P < 0.05; ***P < 0.001; ****P < 0.0001.

To determine whether TIA-1 overexpression can alleviate stress-induced senescence, we investigated senescence markers in HaCaT cells exposed to either NaBu or UV-B irradiation. SA β-gal staining revealed that ectopic expression of TIA-1 significantly reduced the number of SA β-gal-positive cells under both NaBu (Fig. 4a) and UV-B (Fig. 4b) treatment conditions. These results indicate that elevated TIA-1 level decreases the acquisition of SA β-gal expression in response to different stressors. In addition, TIA-1 overexpression markedly attenuated the stress-induced increase in p21 and p16 levels in both NaBu-treated and UV-B-treated cells (Fig. 4c,d), further supporting its role in regulating cellular senescence. Finally, we examined whether TIA-1 overexpression could restore mitophagy activity suppressed by stress. Using the mt-Keima reporter assay, we observed that both treatments reduced FCCP-induced mitophagic flux, as indicated by a decreased red-to-green fluorescence ratio. Importantly, ectopic expression of TIA-1 significantly rescued mitophagy activity under both NaBu and UV-B (Fig. 4e,f) treatment conditions. Together, these results demonstrate that TIA-1 overexpression consistently attenuates stress-induced senescence and restores mitophagy activity in response to senescence-inducing stimuli, underscoring its critical protective role in maintaining mitochondrial quality control under stress conditions.

### TIA-1 overexpression alleviates NaBu-induced mitochondrial dysfunction and limits mtDNA-mediated inflammatory signaling

**Fig. 5:**
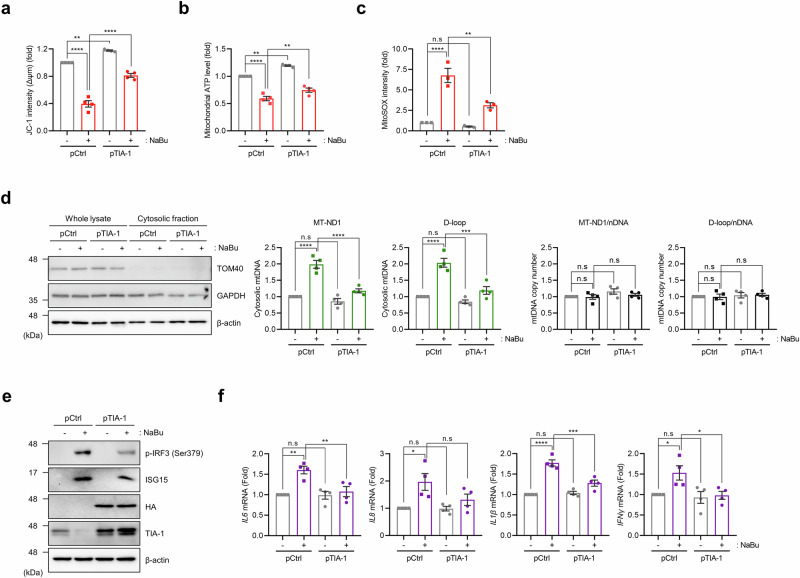
TIA-1 overexpression attenuates mitochondrial dysfunction induced by NaBu treatment. Following ectopic expression of pTIA-1, HaCaT cells were treated with 1 mM sodium butyrate (NaBu) for 48 h. Mitochondrial function was evaluated by measuring membrane potential (Δ*ψ*_m_, JC-1 staining) (part **a**), ATP production (Mitochondrial ToxGlo™) (part **b**), and mitochondrial superoxide levels (MitoSOX™) (part **c**). **d** Cytosolic release of mitochondrial DNA (mtDNA) was evaluated by measuring the levels of mtDNA (ND1 and D-loop) in the cytosolic fraction using quantitative PCR (qPCR). Cytosolic mtDNA (relative level) was calculated as the amount of mtDNA detected in the cytosolic fraction normalized to the total cellular mtDNA copy number measured from whole-cell lysates. Total mtDNA copy number was determined by qPCR using genomic DNA extracted from whole-cell lysates and normalized to the nuclear reference gene *KCNJ10*. TOM40 and GAPDH served as markers for the mitochondrial and cytosolic fractions, respectively, and β-actin was used as a loading control. **e** Levels of IRF3 phosphorylation and free ISG15 were analyzed by western blotting. β-Actin was used as the loading control. **f** *IL6, IL8, IL1β*, and *IFNγ* mRNA levels were analyzed by RT–qPCR. *GAPDH* mRNA was used for normalization. Representative images are shown and data represent mean ± SEM from three independent experiments. IFNγ, interferon-γ; n.s., not significant (*P* > 0.05); *P < 0.05; **P < 0.01; ***P < 0.001.

To further investigate how TIA-1 overexpression modulates cellular responses to stress, we examined its effects on mitochondrial function in NaBu-treated HaCaT cells. NaBu exposure significantly reduced mitochondrial membrane potential, as determined by decreased JC-1 staining intensity. Notably, ectopic expression of TIA-1 significantly preserved mitochondrial membrane potential under NaBu treatment (Fig. 5a). Mitochondrial ATP production assays showed similar results: although NaBu treatment decreased ATP levels, TIA-1 overexpression rescued this decline (Fig. 5b). We also assessed mitochondrial ROS production using MitoSOX staining. NaBu treatment markedly increased ROS levels in HaCaT cells, but TIA-1 overexpression significantly attenuated this stress-induced ROS accumulation (Fig. 5c). Together, these data indicate that TIA-1 overexpression can mitigate NaBu-induced mitochondrial dysfunction.

Because mitophagy is closely linked to the control of cytosolic mtDNA release^29,30^, we next investigated whether stress-induced senescence or TIA-1 knockdown altered mtDNA release. Fractionation and qPCR analysis revealed that both NaBu treatment and TIA-1 knockdown increased cytosolic mtDNA (MT-ND1 and D-loop) levels in HaCaT cells without changing total mtDNA copy number (Supplementary Fig. 3). Importantly, we found that TIA-1 overexpression significantly reduced the relative amount of mtDNA detected in the cytosolic fraction after NaBu treatment, again without altering total mtDNA copy number (Fig. 5d). These findings suggest that TIA-1 overexpression suppresses stress-induced mtDNA release into the cytosol.

Given that cytosolic mtDNA release can activate the cGAS–STING pathway and induce pro-inflammatory gene expression^31^, we examined downstream signaling events. WB analysis showed that NaBu treatment increased phosphorylation of IRF3 and the level of free ISG15 protein, whereas TIA-1 overexpression partially reduced these stress-induced increases (Fig. 5e). Consistently, NaBu treatment upregulated mRNA levels of *IL6*, *IL8*, *IL1β*, and *IFNγ*, whereas TIA-1 overexpression modestly attenuated this transcriptional activation (Fig. 5f). Together, these results demonstrate that TIA-1 overexpression not only protects against NaBu-induced mitochondrial dysfunction but also limits mtDNA-mediated activation of the cGAS–STING inflammatory pathway in senescent HaCaT cells.

### TIA-1 directly associates with the *FUNDC1* mRNA to promote its expression

**Fig. 6:**
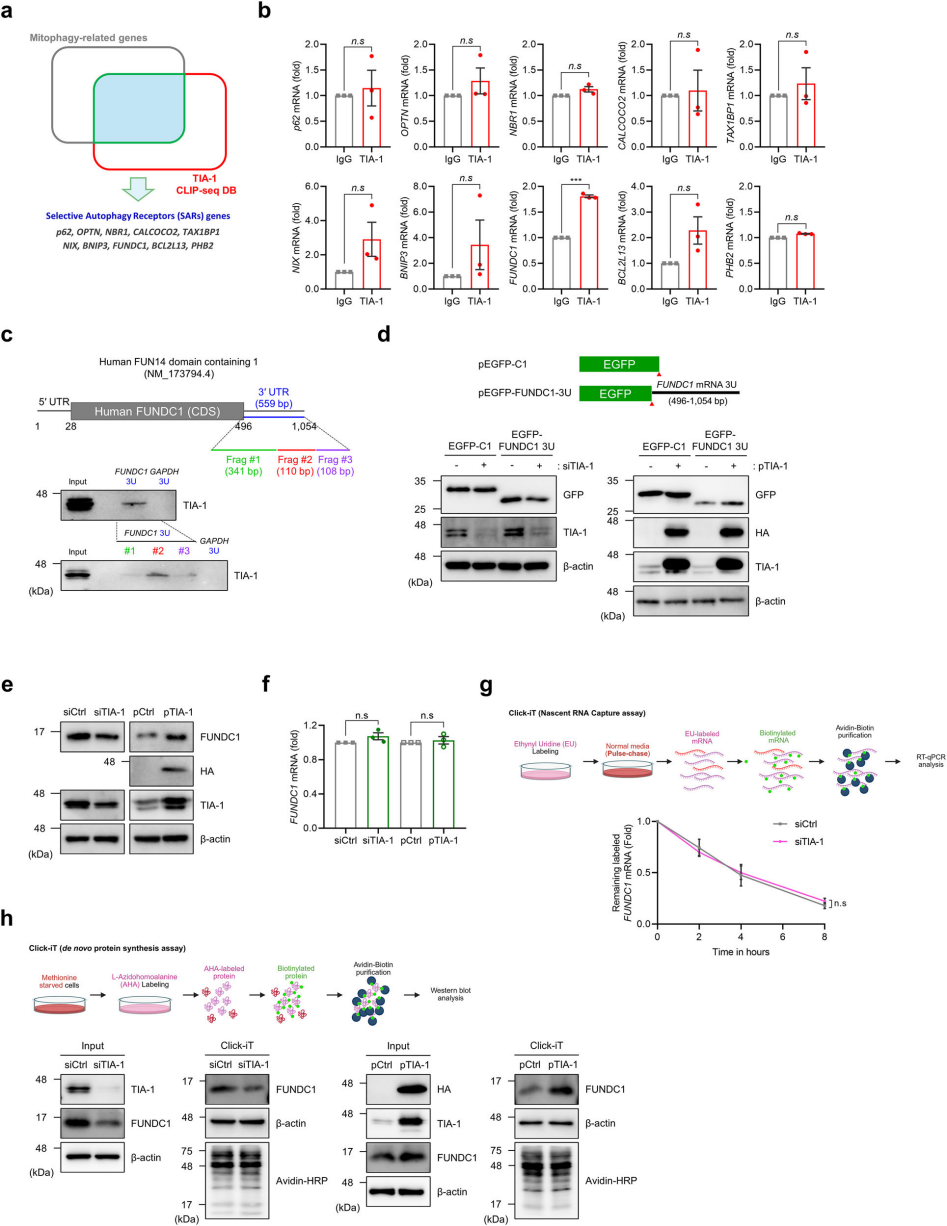
TIA-1 promotes FUNDC1 expression by associating with the 3′UTR of *FUNDC1* mRNA. **a** Schematic diagram illustrating the identification of putative target mRNAs of TIA-1. In silico analysis using TIA-1 crosslinking immunoprecipitation sequencing (CLIP-seq) data combined with GO analysis for mitophagy-related genes identified several SAR genes. **b** The interaction between TIA-1 and SAR gene mRNAs was analyzed by RNP immunoprecipitation followed by RT–quantitative PCR (qPCR) with anti-TIA-1 and normal IgG antibodies. *GAPDH* mRNA was used as the reference gene for normalization. **c** Biotinylated transcripts corresponding to the 3′UTR of *FUNDC1* mRNA (NM_173794.4, denoted as FUNDC1 3U) or control GAPDH 3U were incubated with HaCaT cell lysates, and their association with TIA-1 was determined by biotin pull-down assay. **d** A schematic representation of EGFP reporter constructs containing the 3′UTR of *FUNDC1* mRNA (upper). Reporter expression was analyzed by western blotting (WB) after TIA-1 knockdown or overexpression (bottom). **e**,**f** FUNDC1 expression levels were analyzed using WB and RT–qPCR following TIA-1 knockdown or overexpression. β-Actin served as the loading control for WB, and *GAPDH* mRNA was used as the reference gene for normalization of RT–qPCR data. **g** A brief experimental scheme of Nascent RNA capture assay (upper). Following TIA-1 knockdown for 48 h, the 4 × 10^5^ HaCaT cells were exposed to 4 h EU (0.2 mM) pulse followed by 0, 2, 4, or 8 h chase and analyzed for Click-iT Nascent RNA Capture Kit, as described in the Materials and methods section. Remaining labeled *FUNDC1* mRNA levels were determined by RT–qPCR following normalization to the labeled *GAPDH* mRNA in the same sample (bottom). **h** An experimental scheme of de novo protein synthesis assay (upper). HaCaT cells were exposed to 50 μM AHA (L-azidohomoalanine) for 4 h and analyzed for Click-iT Protein Analysis Detection Kit as described in Materials and methods following TIA-1 knockdown or overexpression. Nascent AHA-labeled proteins were metabolically labeled with biotin using Click-iT reaction buffer. The biotinylated proteins were purified with avidin-magnetic beads and assessed by WB using a streptavidin-horseradish peroxidase conjugate. β-Actin was served as the loading control. Representative images are shown and data represent mean ± SEM from three independent experiments. n.s., not significant (*P* > 0.05); ***P < 0.001.

TIA-1 is an RNA-binding protein that binds to its target mRNAs and can affect their expression by regulating RNA metabolism^16^. To understand the molecular mechanism by which TIA-1 regulates mitophagy, we sought to identify the downstream molecules of TIA-1. By analyzing the TIA-1 crosslinking immunoprecipitation sequencing dataset^32^ and POSTAR3 database^33^, we identified a set of putative TIA-1-bound mRNAs encoding selective autophagy receptors (SARs), which are essential mediators for mitophagy^11,12^ (Fig. 6a). To confirm whether TIA-1 physically associates with these SAR mRNAs, we performed RNP IP using TIA-1 antibody, followed by RT–qPCR. Several SAR mRNAs, including *NIX*, *BNIP3*, *FUNDC1*, and *BCL2L13*, were substantially enriched in TIA-1-containing RNP complexes compared with control IP with IgG, indicating a potential interaction between TIA-1 and these mRNAs (Fig. 6b). Among these candidates, *FUNDC1* mRNA showed statistically significant and consistent enrichment in TIA-1 IP. FUNDC1 (FUN14 domain containing 1) is a known SAR involved in ubiquitin-independent mitophagy^34^ and contains an ~500 nt 3ʹUTR in human (NM_173794.4) (Fig. 6c). To test the interaction between TIA-1 and *FUNDC1* mRNA, we performed in vitro binding assays with biotin-labeled transcripts corresponding to the 3ʹUTR of *FUNDC1* mRNA. TIA-1 bound to the 3ʹUTR of *FUNDC1* mRNA (Fig. 6c), supporting a direct interaction. To further localize the TIA-1-binding region within the *FUNDC1* 3ʹUTR, we next performed a mapping biotinylated RNA pull-down assay. On the basis of reported features of TIA-1 RNA-binding preferences and motif signatures^32,35^, we subdivided the *FUNDC1* 3ʹUTR into three fragments: a 5ʹ segment (region 1), a motif-enriched putative TIA-1-binding segment (region 2), and a 3ʹ segment (region 3) (Supplementary Fig. 4). Biotin-labeled RNA fragments corresponding to each region were generated and subjected to pull-down assays followed by WB for TIA-1. Notably, TIA-1 binding was predominantly detected with region 2, whereas regions 1 and 3 showed markedly weaker binding, indicating that region 2 contains the dominant TIA-1-binding site(s) within the *FUNDC1* 3ʹUTR (Fig. 6c).

To investigate whether TIA-1 modulates FUNDC1 expression via its 3’UTR, we used an EGFP reporter construct containing the *FUNDC1* 3′UTR. TIA-1 knockdown significantly reduced reporter expression, whereas TIA-1 overexpression increased it (Fig. 6d). These results indicate that TIA-1 binds to the 3’UTR of *FUNDC1* mRNA and promotes its expression. Building on these findings, we next examined whether TIA-1 regulates FUNDC1 expression at the endogenous protein level. Consistent with the 3′UTR reporter results, TIA-1 knockdown markedly reduced FUNDC1 protein abundance, whereas TIA-1 overexpression increased FUNDC1 protein levels (Fig. 6e). By contrast, under the same knockdown and overexpression conditions, the protein levels of other TIA-1-bound SAR candidates (BCL2L13, BNIP3, and NIX) were not significantly altered (Supplementary Fig. 5), suggesting that the effect of TIA-1 on mitophagy receptor expression is relatively selective for FUNDC1 in our experimental context. To define the level at which TIA-1 controls FUNDC1 expression, we assessed *FUNDC1* mRNA abundance following TIA-1 knockdown or overexpression. Notably, despite the obvious change in FUNDC1 protein levels, *FUNDC1* mRNA levels did not change significantly (Fig. 6f). Because RNA-binding proteins can modulate target gene expression by altering mRNA decay rates, we next assessed whether TIA-1 affected *FUNDC1* mRNA stability using a nascent RNA capture assay with EU. This analysis revealed no detectable difference in *FUNDC1* mRNA stability upon TIA-1 knockdown (Fig. 6g), indicating that altered mRNA turnover is unlikely to explain the reduction in FUNDC1 protein abundance. Given the dissociation between *FUNDC1* mRNA and protein levels, we next tested whether TIA-1 regulates FUNDC1 expression at the translational level. Using a de novo protein synthesis assay followed by WB, we found that TIA-1 knockdown decreased the newly synthesized FUNDC1 level, whereas TIA-1 overexpression increased it (Fig. 6h), supporting a model in which TIA-1 enhances FUNDC1 expression primarily through translational control.

To address potential concerns regarding siRNA-specific off-target effects, we validated selected key findings using three additional independent TIA-1 siRNAs (siRNAs 2–4), in addition to the primary siRNA used throughout this study (siRNA 1). Despite modest differences in knockdown efficiency and effect size among siRNAs, all four siRNAs consistently reproduced the major findings, including reduced FUNDC1 expression and altered mitochondrial/senescence-related readouts (Supplementary Fig. 6).

### FUNDC1 overexpression partially rescues senescence and mitophagy defects induced by TIA-1 knockdown

**Fig. 7:**
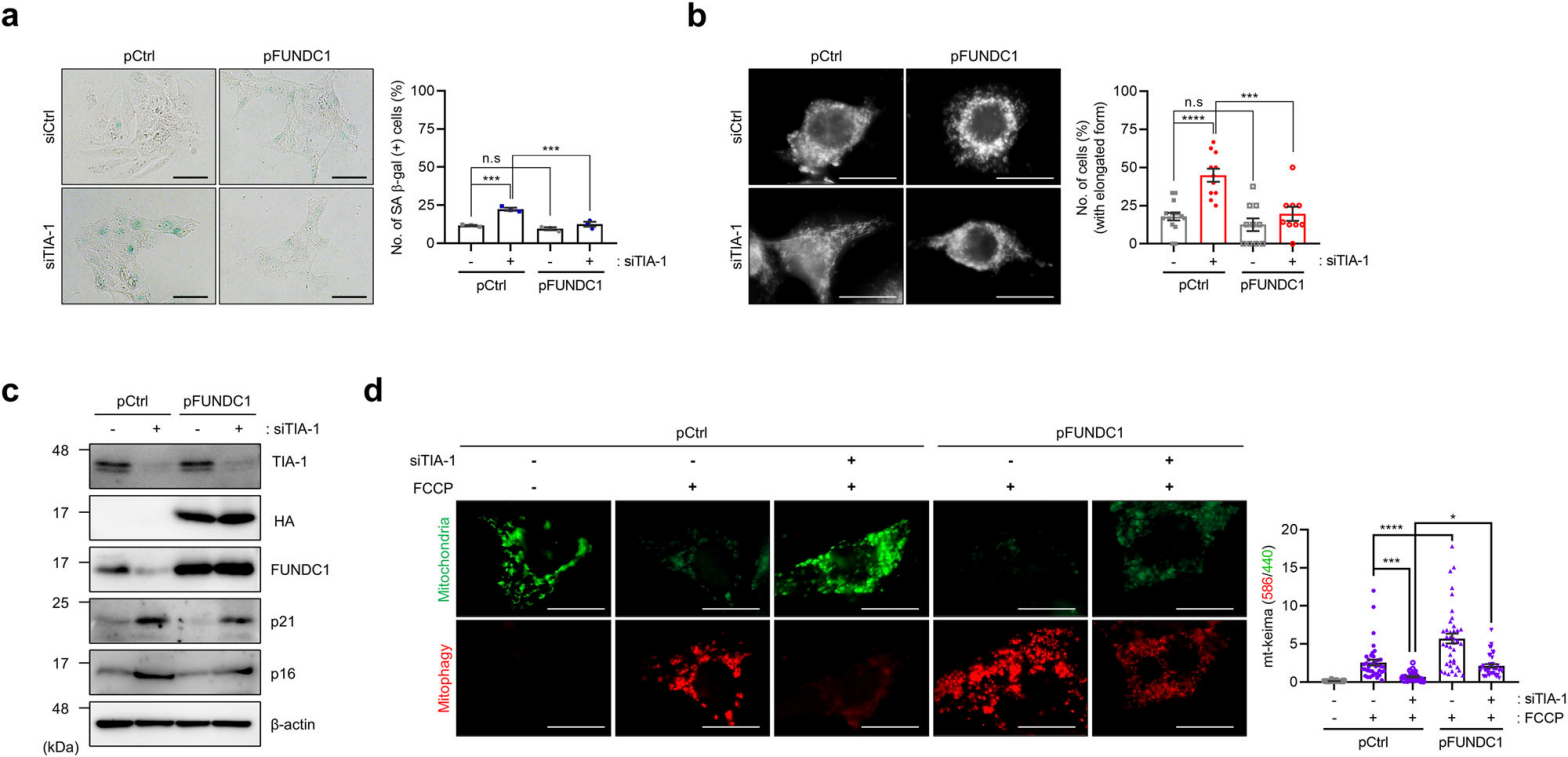
The TIA-1/FUNDC1 axis regulates cellular senescence and mitochondrial dysfunction. Following TIA-1 knockdown, HaCaT cells were transfected with the FUNDC1 overexpressing plasmid for 48 h (parts **a–c**) or 24 h (part **d**). **a** Senescence-associated β-galactosidase (SA β-gal) activity was assessed by X-gal staining (pH 6.0), and the number of SA β-gal-positive cells was quantified. **b** Mitochondrial morphology was analyzed using MitoTracker staining. The number of cells with elongated mitochondria was determined using ImageJ software. **c** Protein expression was analyzed by western blotting. β-Actin was used as the loading control. **d** Mitophagy activity was assessed by monitoring the red fluorescence of the mt-Keima reporter following incubation with FCCP (20 μM), and the fluorescence ratio (red/green) was determined using ImageJ software. Representative images are shown and data represent mean ± SEM from three independent experiments. Scale bar, 20 μm. n.s., not significant (*P* > 0.05); ***P < 0.001 and ****P < 0.0001.

To test whether FUNDC1 mediates the mitochondrial dysfunction and senescence phenotypes caused by TIA-1 depletion, we generated a FUNDC1 overexpression construct by cloning the FUNDC1 coding sequence into an expression vector and confirmed ectopic FUNDC1 expression (Supplementary Fig. 7). We then performed a genetic rescue experiment by overexpressing FUNDC1 in TIA-1 knockdown cells. Consistent with a functional contribution of FUNDC1 downstream of TIA-1, FUNDC1 overexpression significantly reduced the increase in SA β-gal-positive cells induced by TIA-1 KD (Fig. 7a). Likewise, MitoTracker staining showed that FUNDC1 overexpression alleviated the mitochondrial elongation phenotype observed upon TIA-1 KD (Fig. 7b). At the molecular level, FUNDC1 OE partially attenuated the elevated expression of the senescence markers p21 and p16 in TIA-1-depleted cells (Fig. 7c). Finally, in the mt-Keima reporter assay, ectopic expression of FUNDC1 modestly but significantly increased the mt-Keima reporter signal that was reduced by TIA-1 KD (Fig. 7d), indicating partial restoration of mitophagy activity. Collectively, these rescue experiments support a model in which FUNDC1 is a key downstream effector of TIA-1 in regulating mitochondrial morphology, mitophagy, and senescence-associated phenotypes.

### Ectopic expression of TIA-1 restores FUNDC1 expression and promotes mitophagy under stress conditions

**Fig. 8:**
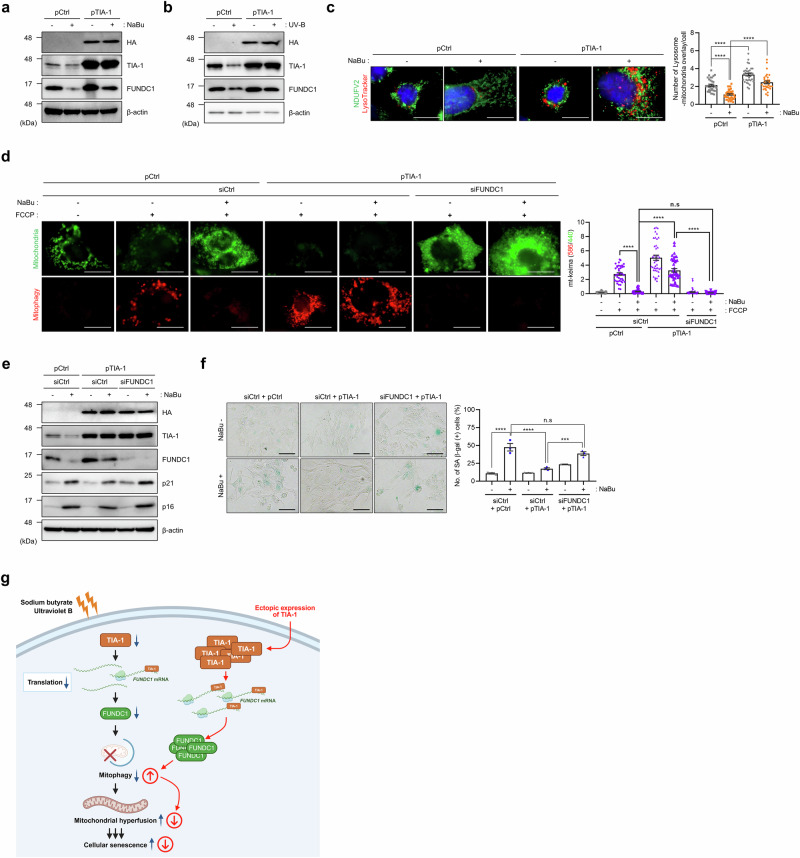
TIA-1 promotes mitophagy and prevents mitochondrial hyperfusion by upregulating FUNDC1. HaCaT cells were transfected with pTIA-1 and further incubated with 1 mM sodium butyrate (NaBu) (part **a**) or exposed to ultraviolet (UV)-B irradiation (75 mJ/cm²) (part **b**). Protein levels were determined by western blotting, and β-actin was used as the loading control. **c** HaCaT cells were transfected with pTIA-1 and further incubated with 1 mM NaBu. Intracellular distribution of mitochondrial NDUFV2 (green) and lysosomes (red) was examined by immunofluorescence microscopy. Nuclei were counterstained with 4′,6-diamidino-2-phenylindole (blue). **d–f** Following FUNDC1 knockdown, HaCaT cells were transfected with TIA-1 overexpressing plasmid for 48 h in NaBu-containing media. **d** Mitophagy activity was assessed by monitoring the red fluorescence signals of the mt-Keima reporter following incubation with FCCP (20 μM), and the fluorescence ratio (red/green) was determined using ImageJ software. **e** Protein expression was analyzed by western blotting. β-Actin was used as the loading control. **f** Senescence-associated β-galactosidase (SA β-gal) activity was assessed by X-gal staining (pH 6.0), and the number of SA β-gal-positive cells was quantified. Representative images are shown, and data represent mean ± SEM from three independent experiments. Scale bar, 20 μm. n.s., not significant (*P* > 0.05); ***P < 0.001; ****P < 0.0001. **g** Schematic model depicting the proposed mechanism: stress-induced downregulation of TIA-1 reduces FUNDC1 expression, leading to impaired mitophagy and mitochondrial hyperfusion, thereby promoting cellular senescence. Ectopic expression of TIA-1 restores FUNDC1 levels, enhances mitophagy, and alleviates mitochondrial dysfunction and senescence.

To further validate the mechanistic link between TIA-1 and FUNDC1-mediated mitophagy under stress, we investigated whether ectopic expression of *TIA-1* (pTIA-1) could restore FUNDC1 expression and mitophagy in HaCaT cells in response to NaBu or UV-B treatment. WB analysis revealed that both stress conditions reduced FUNDC1 protein levels, whereas pTIA-1 significantly rescued FUNDC1 expression under both NaBu (Fig. 8a) and UV-B (Fig. 8b) conditions. We next asked whether pTIA-1 also counteracts the stress-associated impairment of mitophagy at the cellular level. To this end, we performed immunofluorescence analysis using an antibody against the mitochondrial marker NDUFV2 together with LysoTracker to label lysosomes. NaBu treatment decreased the overlap between mitochondrial and lysosomal signals, consistent with reduced mitochondria–lysosome associations under stress. Importantly, pTIA-1 increased mitochondrial–lysosomal colocalization in NaBu-treated cells (Fig. 8c), suggesting that ectopic TIA-1 expression can restore stress-impaired mitophagy, at least in part by promoting mitochondria–lysosome interactions.

To directly test whether the protective effects of pTIA-1 under NaBu exposure are mediated through FUNDC1, we assessed mitophagy and senescence phenotypes in the presence or absence of FUNDC1 knockdown. In the mt-Keima assay, NaBu exposure reduced mt-Keima activity, whereas pTIA-1 modestly but significantly restored it; notably, FUNDC1 knockdown blocked the ability of pTIA-1 to rescue mt-Keima activity under NaBu treatment (Fig. 8d). Consistently, WB analysis showed that pTIA-1 attenuated the NaBu-induced upregulation of p21 and p16, and this effect was partially reversed by FUNDC1 knockdown (Fig. 8e). In line with these molecular changes, SA β-gal staining demonstrated that pTIA-1 reduced the increase in SA β-gal-positive cells induced by NaBu, whereas FUNDC1 knockdown partially abrogated the anti-senescence effect of pTIA-1 (Fig. 8f). Collectively, these results support a model in which ectopic TIA-1 expression restores FUNDC1 levels under NaBu stress, thereby enhancing mitophagy activity and attenuating senescence-associated phenotypes, with FUNDC1 contributing substantially to the protective effects of TIA-1. These findings are summarized in the working model shown in Fig. 8g.

## Discussion

Tight regulation of mitochondrial homeostasis is essential for the maintenance of normal cellular functions^2,10^. Mitochondrial homeostasis is controlled at multiple levels, including biogenesis, dynamics, and mitophagy^10^. Dysregulation of mitochondrial homeostasis leads to mitochondrial dysfunction, which contributes to the pathogenesis of several human diseases^2,5,36^. Mitochondrial dysfunction, as indicated by imbalanced mitochondrial dynamics, decreased membrane potential, and elevated ROS production, has been identified as a significant contributor to cellular senescence^6,23^. In this study, we demonstrated that mitophagy is reduced in stress-induced senescence models, accompanied by decreased expression of TIA-1. We found that TIA-1 knockdown decreased mitophagy, whereas its ectopic expression enhanced mitophagy activity and mitigated senescence marker expression under NaBu or UV-B treatment. These results suggest that TIA-1 may serve as a novel regulator of mitophagy in the context of cellular senescence.

Several studies have shown that senescent cells have highly elongated mitochondria^1,4^. Enhanced elongation of mitochondria has been linked to alterations in mitochondrial dynamics, specifically the downregulation of fission factors such as DRP1, FIS1, and MFF, and the upregulation of fusion factors such as MFN1/2 and OPA1. Changes in the expression levels of these proteins have been consistently observed in senescent cells^7–9^. Furthermore, the reduction in mitophagy activity, which is crucial for the removal of damaged mitochondria, may contribute to the excessive elongation of mitochondria in senescent cells^13,26,37^. Our previous research documented reduced MFF expression in senescent HaCaT cells^9^, and in this study, we further identified a reduction in FUNDC1 expression. Importantly, we have shown that TIA-1 has a key role in regulating the expression of MFF and FUNDC1 in senescent cells. These findings underscore the central role of TIA-1 in maintaining mitochondrial homeostasis and highlight its potential as a therapeutic target for diseases caused by mitochondrial dysfunction, including cancer and age-related diseases.

Notably, although mitochondrial elongation (hyperfusion) and impaired mitophagy co-occur in our system, the directionality of this relationship remains to be fully established. Long-term lysosomal inhibition with bafilomycin A1 was sufficient to induce a time-dependent increase in mitochondrial elongation in otherwise untreated cells, consistent with the possibility that reduced mitophagic flux can promote hyperfusion-like remodeling. Conversely, forced mitochondrial fission via DRP1 or MFF overexpression modestly enhanced mt-Keima activity in control cells but did not rescue the mitophagy defect in TIA-1-depleted cells, suggesting that altered fusion–fission balance alone is unlikely to account for the mitophagy impairment and that additional TIA-1-dependent steps are required for effective mitochondrial quality control. These additional observations are presented in Supplementary Fig. 8 and underscore the need for future studies to define how TIA-1 coordinates mitochondrial homeostasis under stress.

Despite its critical role in regulating mitochondrial homeostasis and cellular senescence, much remains unknown about the molecular functions of TIA-1 and the mechanisms governing its expression. As an RNA-binding protein, TIA-1 modulates RNA metabolism processes such as alternative splicing and translation^16^. In response to various stress stimuli, it regulates the expression of its target mRNAs by forming stress granules or interacting with other proteins^38^. Mutations in the *TIA-1* gene have been associated with several human diseases such as amyotrophic lateral sclerosis and myopathy, highlighting its clinical significance^19,39^. Although certain factors, including *microRNA-30-5p* (*miR-30-5p*), have been identified as regulators of TIA-1 expression by our group^9^, the precise mechanisms controlling TIA-1 expression and activity remain largely unclear. Notably, how TIA-1 expression is diminished during cellular senescence is a question that requires further investigation. Our findings demonstrated that TIA-1 expression is reduced following NaBu treatment (Fig. 1). Exploring how HDAC inhibitor butyrate mediates this downregulation could provide valuable insights into the regulatory mechanisms underlying TIA-1 expression. In this regard, prior work has suggested that NaBu can repress TIA-1 transcription via an Sp1-dependent mechanism^40^, raising the possibility that transcriptional suppression contributes to the decrease in TIA-1 under butyrate stress. Additionally, given our recent finding that the *miR-30-5p* family negatively regulates TIA-1 expression^9^, it will be important to determine whether butyrate-induced senescence also involves microRNA-mediated downregulation of TIA-1. Together, these potential transcriptional and post-transcriptional mechanisms represent plausible routes by which butyrate may reduce TIA-1 levels and warrant further investigation in future studies.

To extend the relevance of the TIA-1/FUNDC1 axis beyond immortalized cell lines, we examined publicly available transcriptomic datasets. Across multiple independent GEO series, TIA-1 and FUNDC1 exhibited coordinated decreases across diverse tissues and/or conditions, consistent with a positive correlation between these genes in vivo-relevant contexts (Supplementary Fig. 9a). In addition, in primary keratinocytes undergoing replicative senescence, TIA-1 and FUNDC1 protein levels declined in parallel as senescence progressed (Supplementary Fig. 9b), supporting the physiological plausibility of a TIA-1–FUNDC1 relationship in senescence. Future studies in aged tissues and in vivo models will be required to define the tissue specificity, upstream drivers, and functional consequences of this axis during aging and stress responses.

FUNDC1 is a mitochondrial receptor that directly interacts with LC3 and mediates a PINK1/Parkin-independent pathway of mitophagy in response to various stimuli^20,41^. Dysregulation of mitophagy due to alterations in FUNDC1 expression has been implicated in several pathological conditions, including cancer, fibrosis, and cardiovascular disease^34,42–44^. In this study, we observed the downregulation of FUNDC1 accompanied by a reduction in mitophagy in the NaBu-induced senescence model. In addition to its role in regulating mitophagy, FUNDC1 has a critical role in maintaining mitochondrial homeostasis by contributing to the formation of mitochondrial-endoplasmic reticulum contact sites and by influencing mitochondrial biogenesis and mitochondrial dynamics^45,46^. Therefore, a detailed understanding of FUNDC1 expression and its regulatory mechanisms is important for advancing our knowledge of FUNDC1-mediated pathology. Although our study has identified TIA-1 as a positive regulator of FUNDC1 expression, we note that TIA-1 also showed a potential to associate with the mRNAs of other mitophagy receptors, including NIX, BNIP3, and BCL2L13. However, the protein levels of these receptors were not significantly altered by TIA-1 knockdown or overexpression, supporting a model in which TIA-1 preferentially regulates FUNDC1 rather than broadly modulating multiple mitophagy receptors under our experimental conditions (Fig. 6 and Supplementary Fig. 5). At the same time, we cannot exclude the possibility that TIA-1 regulates additional SAR transcripts not examined here or that such regulation is stimulus-dependent or context-dependent. Given prior reports that FUNDC1 also participates in mitochondrial fission^20^, together with our findings, it is plausible that TIA-1 contributes to mitochondrial quality control by coordinating mitochondrial fission (via MFF and FUNDC1) and mitophagy through selective regulation of FUNDC1 expression. Further investigations will be required to identify additional regulatory factors and to elucidate the pathways governing FUNDC1 function across multiple levels, including transcriptional, post-transcriptional, and post-translational regulation, as well as protein–protein interactions. These efforts will contribute to a more comprehensive understanding of FUNDC1-mediated mitochondrial homeostasis.

In conclusion, our findings provide new insights into the regulatory roles of TIA-1 and FUNDC1 in mitochondrial homeostasis and cellular senescence. By identifying TIA-1 as a novel regulator of FUNDC1 expression and highlighting its impact on mitophagy, this study underscores the importance of these factors in maintaining mitochondrial structure and functionality. These results not only advance our understanding of the molecular mechanisms underlying mitochondrial dysregulation in the stress-induced senescence model but also lay the groundwork for future investigations into potential therapeutic strategies targeting mitochondrial dysfunction in cellular senescence and age-related and pathological conditions.

## Supplementary information

Supplementary Information

## Data Availability

The data analyzed during this study are included in this published article and the supplemental data files.
