# Supplementary material for: TIA-1 promotes FUNDC1-mediated mitophagy to protect against stress-induced cellular senescence
Source: Exp Mol Med. 2026 Jun 5;58(6):1940–52. doi: 10.1038/s12276-026-01752-w (PMC13324547; doi:10.1038/s12276-026-01752-w)
Supplement: Supplementary file 1 — Supplementary Information [file 12276_2026_1752_MOESM1_ESM.pdf]

**Supplementary materials**

# TIA-1 promotes FUNDC1-mediated mitophagy to protect against stress-induced cellular senescence

Seongho Cha[1, 2], Myeongwoo Jung[1], Hyosun Tak[3], Seungyeon Ryu[1,2], Sukyoung Han[1,2], Dongwoo Chae[4], Jiyoon Kim[2, 5, 6], Seung Min Jeong[1, 2, 6], Wook Kim[7], and Eun Kyung Lee[1, 2, 6, *]

[1] Department of Biochemistry, College of Medicine, The Catholic University of Korea, Seoul 06591, South Korea

[2] Department of Medical Science, Graduate School, The Catholic University of Korea, Seoul 06591, South Korea

[3] INSERM U1052, CNRS UMR-5286, Cancer Research Center of Lyon (CRCL), Lyon 69008, France

[4] Department of Pharmacology, Yonsei University College of Medicine, Seoul 03722, South Korea

[5] Department of Pharmacology, College of Medicine, The Catholic University of Korea, Seoul 06591, South Korea

[6] Institute for Aging and Metabolic Diseases, College of Medicine, The Catholic University of Korea, Seoul 06591, South Korea

[7] Department of Molecular Science & Technology, Ajou University, Suwon 16499, South Korea

[*] Correspondence should be addressed to Eun Kyung Lee; leeek@catholic.ac.kr

## Materials and Methods

*Primary keratinocyte culture*

Anonymized human primary foreskin keratinocytes were purchased from Invitrogen™ (Waltham, MA, USA) and cultured in KGM® Gold Keratinocyte Growth Medium BulletKit® (Lonza Bioscience™, Basel, Switzerland) containing 10μM Y-27632 (ROCK inhibitor) (Tocris Bioscience, Bristol, UK) at 37℃. All procedures were conducted with approval from the Institutional Review Board of The Catholic University of Korea (IRB No. MC25ZASIOO71).

To induce replicative senescence, human primary keratinocytes were passaged until they became senescent. Primary keratinocytes at population doubling level 2 (PDL2) were used as young cells and primary keratinocytes at PDL10 were used as senescent cells.

*In silico analysis of the GSE dataset*

Gene expression profiling data sets were obtained from the National Center of Biotechnology Information (NCBI) Gene Expression Omnibus (GEO) database. GSE11882, GSE38718, GSE237029, GSE67827, GSE58915, and GSE160841 sets were used to analyze *TIA-1* and *FUNDC1* mRNA. Pearson correlation analysis was performed to evaluate the association between *TIA-1* and *FUNDC1* expression in each dataset.

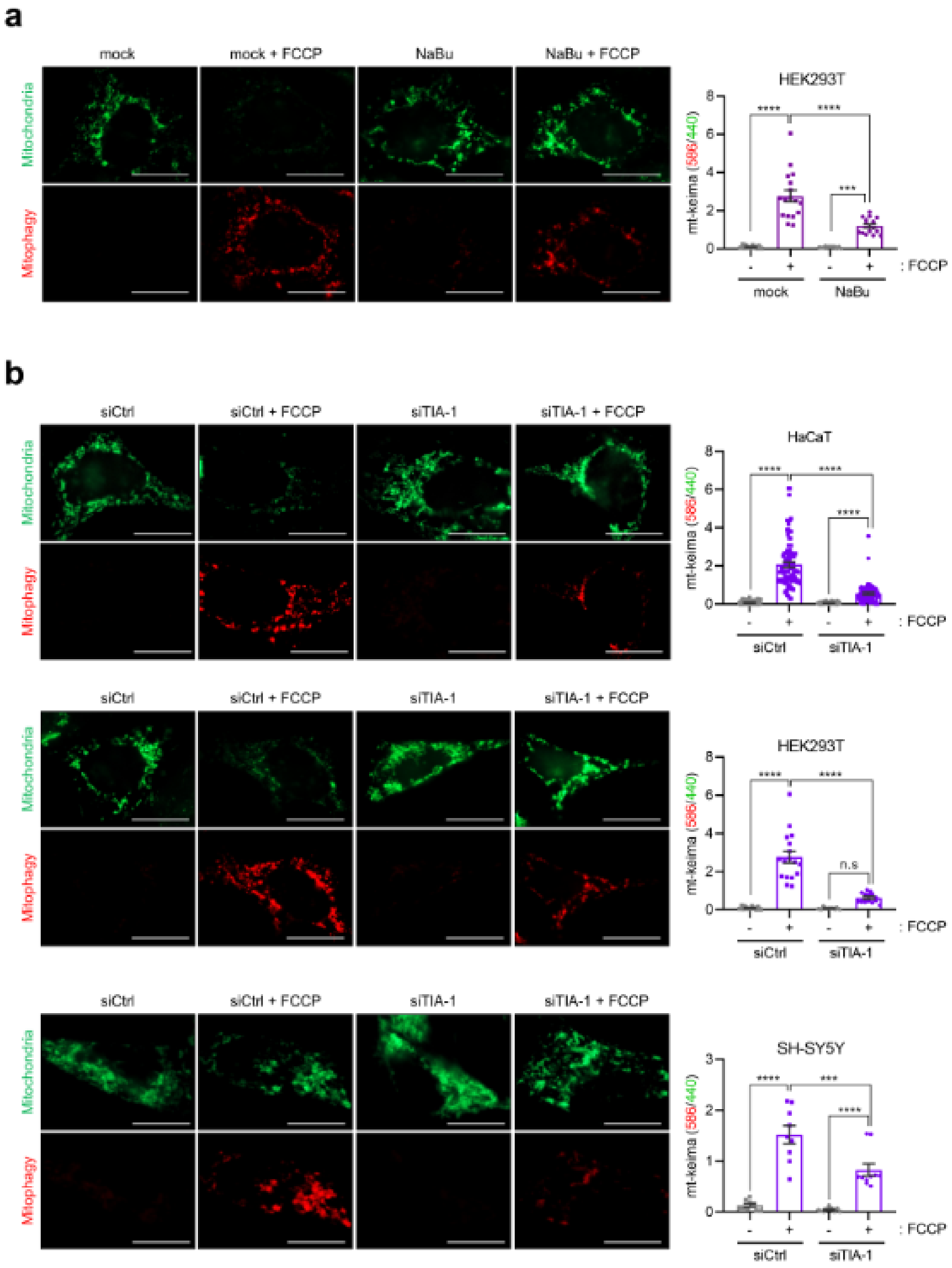


**Supplementary Fig. 1. Mitophagy activity assessed by mt-Keima reporter assay**

(a and b) HEK293T cells transfected with mt-Keima were incubated with 1 mM sodium butyrate (NaBu) for 72 hours (a) or HaCaT, HEK293T, and SH-SY5Y cells were co-transfected with mt-Keima and siRNAs (siCtrl or siTIA-1), followed by 48 hours of incubation (b). To induce mitophagy, cells were

treated with FCCP (20 μM) and oligomycin (5 μM), and mitophagy activity was assessed by quantifying fluorescence signals (red/green) from the mt-Keima reporter using ImageJ software. Representative images are shown, and data are presented as mean ± SEM from three independent experiments. Scale bar, 20 μm. n.s., not significant ($p > 0.05$); ***, $p < 0.001$; ****, $p < 0.0001$.

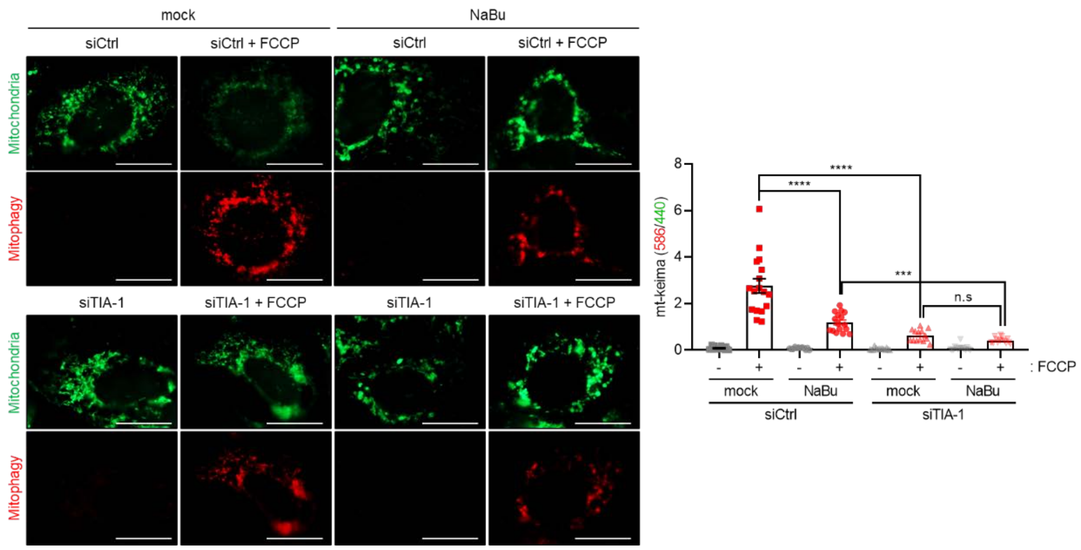


**Supplementary Fig. 2. Mitophagy activity assessed by mt-Keima reporter assay in HEK293T cells**

Following TIA-1 knockdown, HEK293T cells were treated with 1 mM NaBu for 48 hours. Mitophagy activity was assessed by monitoring the red fluorescence signals of the mt-Keima reporter following treatment with FCCP (20 μM), and the fluorescence ratio (red/green) was determined using ImageJ software. Representative images are shown and data represent mean ± SEM from three independent experiments. Scale bar, 20 μm. n.s., not significant ($p > 0.05$); ***, $p < 0.001$; ****, $p < 0.0001$.

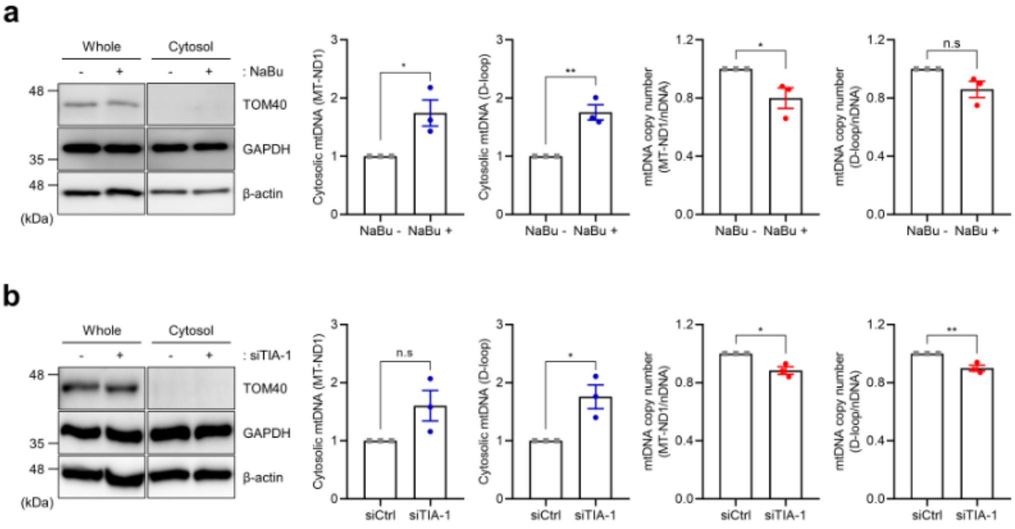


**Supplementary Fig. 3. Release of mitochondrial DNA after NaBu treatment or TIA-1 knockdown**

(a and b) HaCaT cells were incubated with the media containing 1 mM sodium butyrate (NaBu) for 72 hours (a) or transfected with siRNAs (siCtrl or siTIA-1) for 48 hours (b). Fractions were confirmed by WB analysis. TOM40 and GAPDH served as markers for the mitochondrial and cytosolic fraction, respectively, and β-actin was used as a loading control. Cytosolic release of mitochondrial DNA (mtDNA) was evaluated by measuring the levels of mtDNA (ND1 and D-loop) in the cytosolic fraction using qPCR. Cytosolic mtDNA (relative level) was calculated as the amount of mtDNA detected in the cytosolic fraction normalized to the total cellular mtDNA copy number measured from whole-cell lysates. Total mtDNA copy number was determined by qPCR using genomic DNA extracted from whole-cell lysates and normalized to the nuclear reference gene *KCNJ10*. Representative images are shown. Data are presented as mean ± SEM from three independent experiments. n.s., not significant ($p > 0.05$); *$p < 0.05$; **$p < 0.01$.

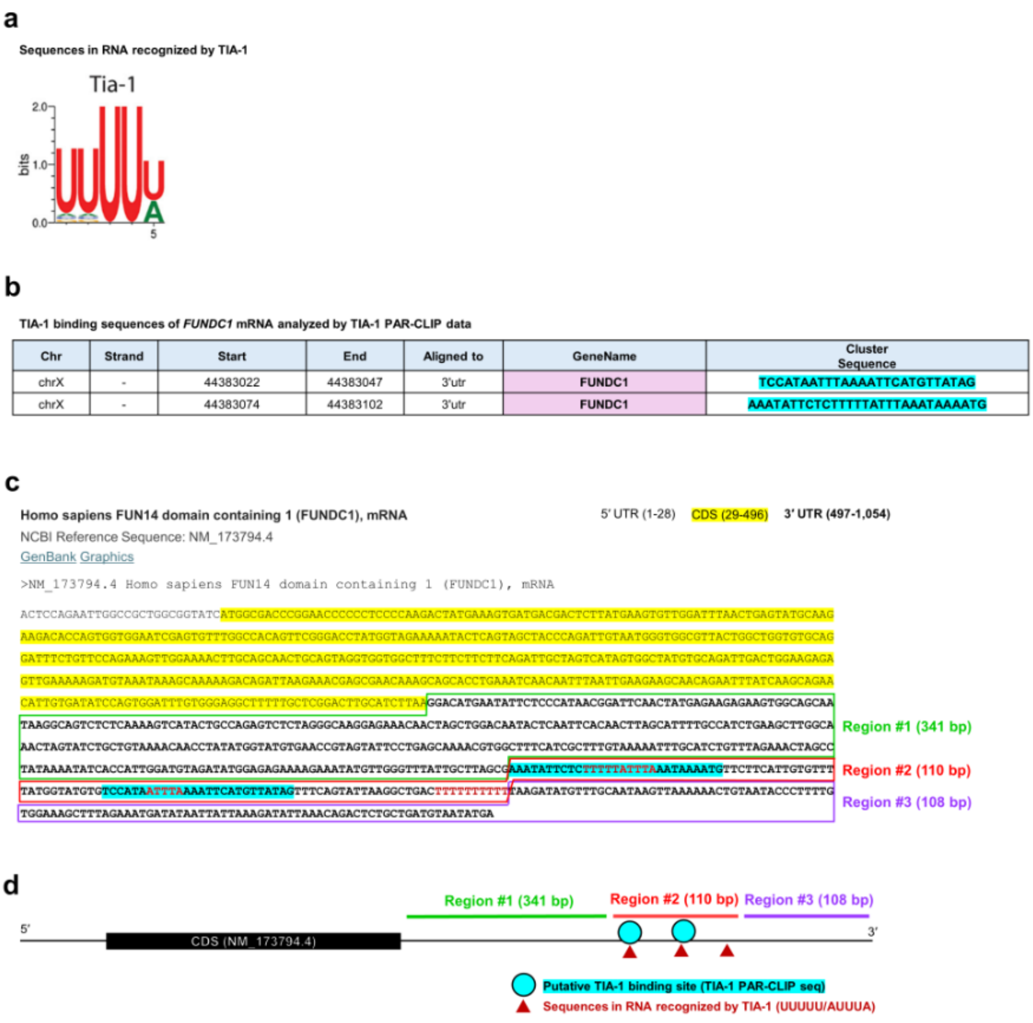


**Supplementary Fig. 4. Schematic of the three 3′UTR fragments used for the biotin pull-down assay**

(a) Sequence motif representing the most frequent nucleotide in target mRNA recognized by TIA-1 [1]. (b) PAR-CLIP–identified clusters of TIA-1 binding sites within the 3′ UTR of *FUNDC1* mRNA. Chromosomal location (Chr), strand orientation, start and end positions, aligned genomic region, gene name, and cluster sequences are shown. The gene name is highlighted in pink, and the cluster sequences are highlighted in blue [2]. (c) Schematic representation of the *FUNDC1* mRNA sequence (NM_173794.4). The 5′ UTR and coding sequence (CDS) regions are indicated. The 3′ UTR was divided into three fragments. Region #2 (110 bp; red box) represents the putative TIA-1 binding region containing both a predicted TIA-1 binding motif (indicated in deep red/light red) and TIA-1 binding

sequences identified from PAR-CLIP data (highlighted in blue). Region #1 (341 bp; green box) and Region #3 (108 bp; purple box) correspond to the upstream and downstream regions of Region #2 within the 3′ UTR of *FUNDC1* mRNA. (d) A schematic representation of the subdivided *FUDNC1* 3'UTR fragments: the 5′ portion (Region #1), the predicted motif-containing segment (Region #2), and the 3′ portion (Region #3).

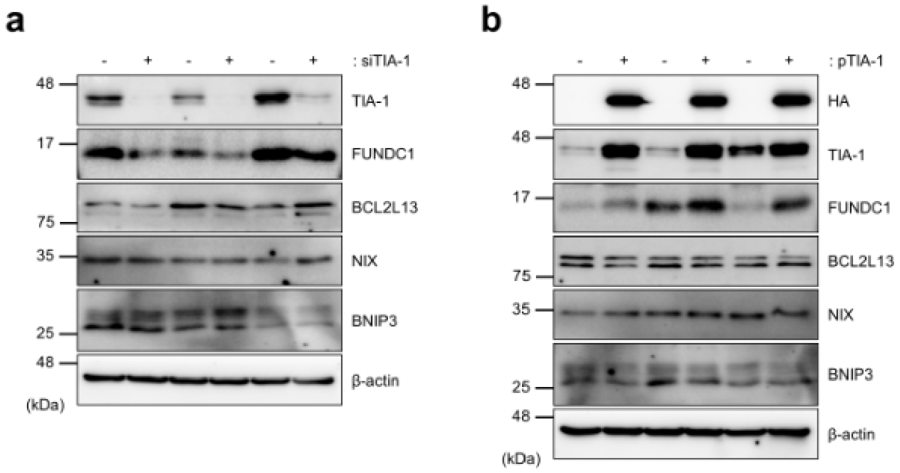


**Supplementary Fig. 5. Relative expression of several selective autophagy receptors (SARs) in HaCaT cells following TIA-1 regulation**

(a and b) HaCaT cells were transfected with siRNAs (siCtrl or siTIA-1) (a) or plasmids (pCtrl or pTIA-1) (b). Relative protein expression of several SARs (BCL2L13, NIX, and BNIP3) was analyzed using WB. β-actin served as the loading control for WB. Representative images are shown from three independent experiments.

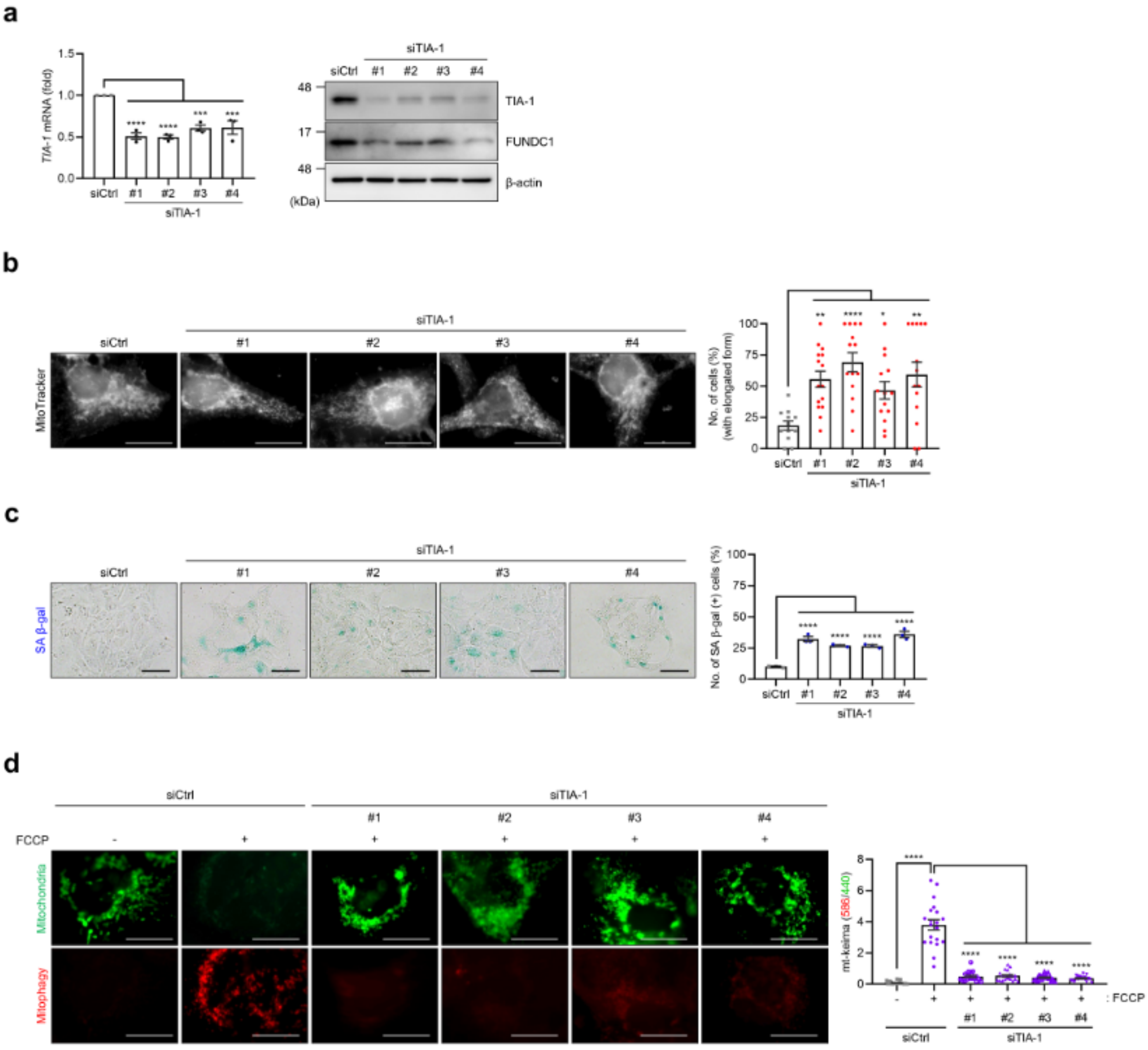


**Supplementary Fig. 6. Multiple independent siRNAs against TIA-1 consistently regulate FUNDC1 expression, cellular senescence, and mitophagy**

Following transfection of multiple siRNAs against TIA-1 (#1; original TIA-1 siRNA sequence used throughout this study, #2–#4; three additional independent siRNAs targeting TIA-1) for 72 hours (a-c) or 48 hours (d), (a) *TIA-1* mRNA levels were analyzed using RT-qPCR and protein levels were determined using WB. β-actin served as the loading control for WB, and *GAPDH* mRNA was used as the reference gene for normalization of RT-qPCR data. (b) Mitochondrial morphology was analyzed using MitoTracker staining. The number of cells with elongated mitochondria was determined using ImageJ software. (c) SA β-gal activity was assessed by X-gal staining (pH 6.0) and the number of SA

β-gal-positive cells was quantified. (d) Mitophagy activity was assessed by monitoring the red fluorescence signals of the mt-Keima reporter following treatment with FCCP (20 μM), and the fluorescence ratio (red/green) was determined using ImageJ software. Representative images are shown. Data are presented as mean ± SEM from three independent experiments. Scale bar, 20 μm. *$p < 0.05$; **$p < 0.01$; ***$p < 0.001$; ****$p < 0.0001$.

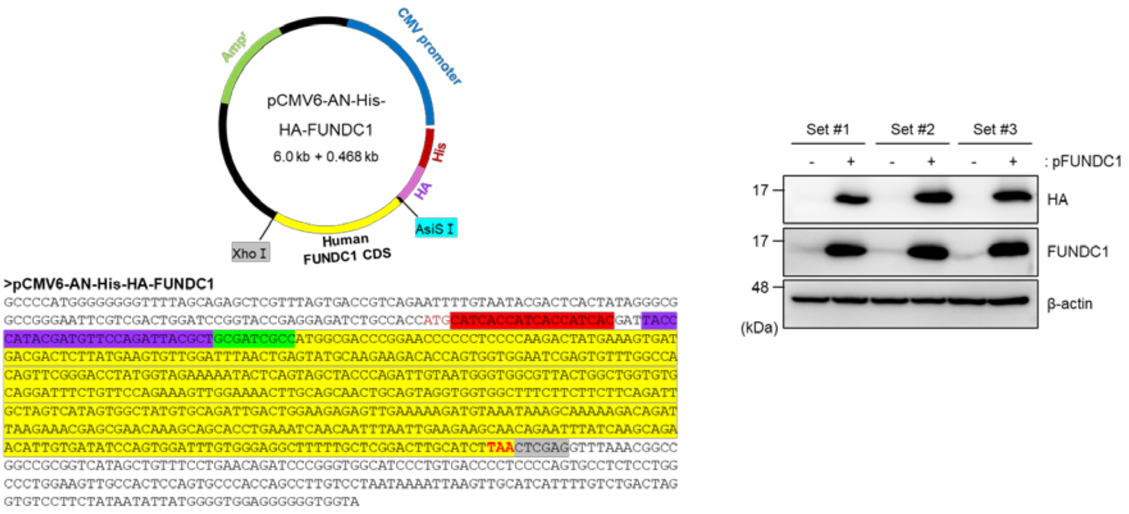


**Supplementary Fig. 7. Construct of HA-tagged FUNDC1 overexpression vector**

(Left) Schematic representation of the FUNDC1 overexpression plasmid map and sequence containing the CMV promoter, His and HA tags, human *FUNDC1* CDS without 3′ UTR of its mRNA. (Right) Expression of HA-tagged FUDNC1 was analyzed by WB. β-actin served as the loading control. Representative images are shown from three independent experiments.

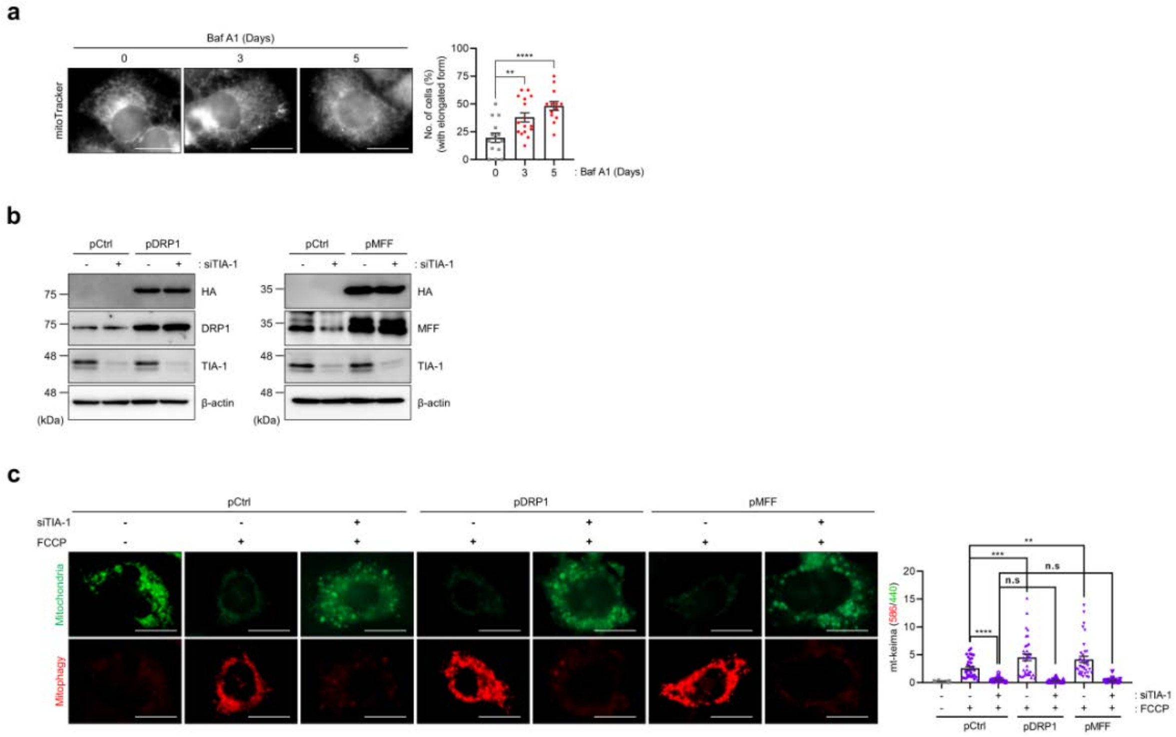


**Supplementary Fig. 8. Pharmacological mitophagy inhibition causes mitochondrial hyperfusion and forced mitochondrial fission partially elevates mitophagy**

(a) HaCaT cells were treated with 20 nM Bafilomycin A1, followed by incubation for 0, 3, or 5 days. Mitochondrial morphology was analyzed using MitoTracker staining. The number of cells with elongated mitochondria was determined using ImageJ software. (b and c) Following TIA-1 knockdown, HaCaT cells were transfected with DRP1 or MFF overexpressing plasmids for 24 hours. (b) Protein expression was analyzed by WB. β-actin was used as the loading control. (c) Mitophagy activity was assessed by monitoring the red fluorescence signals of the mt-Keima reporter following incubation with FCCP (20 μM) and the fluorescence ratio (red/green) was determined using ImageJ software. Representative images are shown and data represent mean ± SEM from three independent experiments. Scale bar, 20 μm. n.s., not significant ($p > 0.05$); **$p < 0.01$; ***$p < 0.001$; ****$p < 0.0001$.

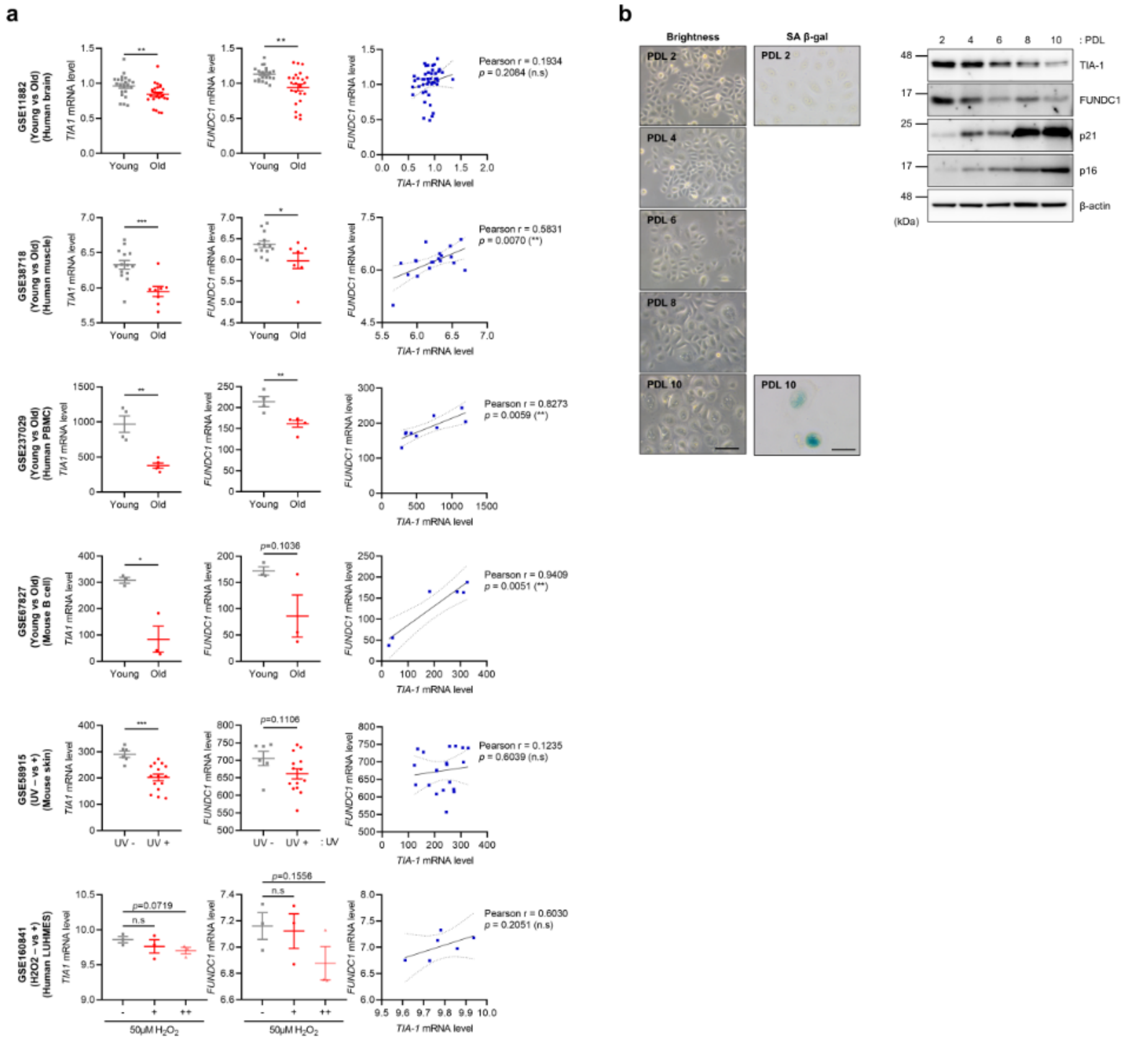


**Supplementary Fig. 9. Downregulation of *TIA-1* and *FUNDC1* expressions in aged tissues and replicative senescent human primary keratinocytes**

(a) Relative levels of *TIA-1* and *FUNDC1* mRNA in several Gene Expression Omnibus (GEO) datasets (GSE11882, GSE38718, GSE237029, GSE67827, GSE58915, and GSE160841). Pearson correlation analysis was performed to evaluate the association between *TIA-1* and *FUNDC1* expression within each dataset. (b) Cell morphology (left), SA β-gal images (middle), and TIA-1 and FUDNC1 protein levels (right) of serially passaged human primary keratinocytes. For SA β-gal analysis, cells were incubated with a staining solution (pH 6.0), and the number of SA β-gal-positive cells was counted. Protein expression was determined by Western blotting (WB) and β-actin was used as the loading control.

Representative images are shown and data represent a single experiment. Scale bar, 20 μm. n.s., not significant ($p > 0.05$); *$p < 0.05$; **$p < 0.01$; ***$p < 0.001$.

**Supplementary Table 1. Oligonucleotide sequences used in this study**

| For RT-qPCR | Forward primers (5′ → 3′) | Reverse primers (5′ → 3′) |
|---|---|---|
| *TIA-1* | CGAGATGCCCAAGACTCTATACG | CCTTACCCATTATCTTCCGTCCA |
| *GAPDH* | AGGTCGGTGTGAACGGATTTG | TGTAGACCATGTAGTTGAGGTCA |
| *p62* | GACTACGACTTGTGTAGCGTC | AGTGTCCGTGTTTCACCTTCC |
| *OPTN* | AAAGAGCGTCTAATGGCCTTG | GTTCAGACACGATGCCCAACA |
| *NBR1* | AGGAGCAAAACGACTAGCTGC | TCTGGGGTCTTCATGTCTGAT |
| *CALCOCO2* | ATTTCATCCCTCGTCGAAAGGA | TAGAGGTGTAATACTCACGGGTT |
| *TAX1BP1* | GCAGACCTGCATACTGCAC | GCCATCTGAAGACGGAGTTTC |
| *NIX* | TTGGATGCACAACATGAATCAGG | TCTTCTGACTGAGAGCTATGGTC |
| *BNIP3* | CAGGGCTCCTGGGTAGAACT | CTACTCCGTCCAGACTCATGC |
| *FUNDC1* | CCTCCCCAAGACTATGAAAGTGA | AAACACTCGATTCCACCACTG |
| *BCL2L13* | TGGTTTTGCTACGACAAATGCT | TGCCGAATAGTCCTCCAGGT |
| *PHB2* | GTGCGCGAATCTGTGTTCAC | GATAATGGGGTACTGGAACCAAG |
| *MT-ND1* | GAACTAGTCTCAGGCTTCAACATCG | CTAGGAAGATTGTAGTGGTGAGGGTG |
| *MT-D-loop* | CATAAAGCCTAAATAGCCCACACG | CCGTGAGTGGTTAATAGGGTGATA |
| *KCNJ10* | GCGCAAAAGCCTCCTCATT | CCTTCCTTGGTTTGGTGGG |
| *IL6* | CAGGAGCCCAGCTATGAACT | GAAGGCAGCAGGCAACAC |
| *IL8* | GAGTGGACCACACTGCGCCA | TCCACAACCCTCTGCACCCAGT |
| *IL1β* | AGCTACGAATCTCCGACCAC | CGTTATCCCATGTGTCGAAGAA |
| *IFNγ* | TCGGTAACTGACTTGAATGTCCA | TCGCTTCCCTGTTTTAGCTGC |
| **For cloning** | **Forward primers (5′ → 3′)** | **Reverse primers (5′ → 3′)** |
| pEGFP-FUNDC1 3U | AAAAAGATCTTAAGGACATGAATATTC TCCCATA | AAAAGAATTCTCATATTACATCAGCAG AGTC |
| phmt-Keima | AAAAGCTAGCATGTCCGTCCTGACGCC G | AAAACTCGAGTTAGCCCAGCAGGGAG TG |
| pHA-FUNDC1 | AAAAGCGATCGCCATGGCGACCCGGA ACCCC | AAAACTCGAGTTAAGATGCAAGTCCG AGCA |
| pHA-DRP1 | AAAAGCGATCGCCATGGAGGCGCTAAT TCCT | AAAACTCGAGTCACCAAAGATGAGTC TCCC |
| **For BPD** | **Sense sequences (5′ → 3′)** | **Antisense sequences (5′ → 3′)** |
| FUNDC1 3U | CCAAGCTTCTAATACGACTCACTATAG GGAGAGGACATGAATATTCTCCCATA | TCATATTACATCAGCAGAGTC |
| FUNDC1 3U Region #1 | CCAAGCTTCTAATACGACTCACTATAG GGAGAGGACATGAATATTCTCCC | CGCTAAGCAATAAACCCA |

| | | |
|---|---|---|
| FUNDC1 3U Region #2 | CCAAGCTTCTAATACGACTCACTATAG GGAGATGCTTAGCGAAATATTCT | GCAAACATATCTTAAAAA |
| FUNDC1 3U Region #3 | CCAAGCTTCTAATACGACTCACTATAG GGAGATAAGATATGTTTGCAATA | TCATATTACATCAGCAGA |

| siRNA | Sense sequences (5′ → 3′) | Antisense sequences (5′ → 3′) |
|---|---|---|
| Control siRNA (siCtrl) | AAUUCUCCGAACGUGUCACGUUU | ACGUGACACGUUCGGAGAAUUU |
| siTIA-1 #1 | AACACAACAAAUUGGCCAGUAUU | UACUGGCCAAUUUGUUGUGUU |
| siTIA-1 #2 | GCAAGUUCCUGCAUAUGGAAUGUAU UU | AUACAUUCCAUAUGCAGGAACUUGC UU |
| siTIA-1 #3 | AGAAUAUCAGAUGCCCGAGUGGUAU U | UACCACUCGGGCAUCUGAUAUUCUU U |
| siTIA-1 #4 | GGCAACAGGAAAGUCUAAGGGAUAU UU | AUAUCCCUUAGACUUUCCUGUUGCCU U |
| siFUNDC1 | GCAGCACCUGAAAUCAACAUU | UGUUGAUUUCAGGUGCUGCUU |

## Supplementary Table 2. Antibodies information used in this study

| Antibodies | Company | Catalog number |
|---|---|---|
| TIA-1 | Cell Signaling Technology, Inc. (Danvers, MA, USA) | #86050 |
| p21 | | #2947 |
| GAPDH | | #2118 |
| p-IRF3 | | #79945 |
| ISG15 | | #2743 |
| BCL2L13 | | #61974 |
| NIX | | #12396 |
| BNIP3 | | #44060 |
| MFF | | #84580 |
| GFP | Santa Cruz Biotechnology, Inc. (Dallas, TX, USA) | #sc-9996 |
| Lamin B | | #sc-6216 |
| TOM40 | | #sc-11414 |
| p16 | | #sc-1661 |
| FUNDC1 | Abcam (Plc., Cambridge, UK) | #ab224722 |
| NDUFV2 | Proteintech (Rosemont, IL, USA) | #15301-1-AP |
| HA | BioLegend (San Diego, CA, USA) | #901501 |
| DRP1 | BD Transduction Laboratories™ (Franklin Lakes, NJ, USA) | #611112 |

| | | |
|---|---|---|
| Streptavidin-HRP | Invitrogen™<br>(Waltham, MA, USA) | #S911 |
| β-actin | Genetex, Inc.<br>(Irvine, CA, USA) | #GTX629630 |
| Anti-mouse HRP-conjugated secondary Ab<br>Anti-Rabbit HRP-conjugated secondary Ab<br>Anti-Goat HRP-conjugated secondary Ab<br>Normal Rabbit IgG | Sigma-Aldrich<br>(Burlington, MA, USA) | #AP124P<br>#AP132P<br>#AP186P<br>#12-370 |
| Anti-Rabbit IgG H&L (Alexa Fluor® 488) | Abcam<br>(Plc., Cambridge, UK) | #ab150077 |

## References

[1] Podszywalow-Bartnicka P, Neugebauer KM. Multiple roles for AU-rich RNA binding proteins in the development of haematologic malignancies and their resistance to chemotherapy. RNA Biol. 2024 Jan;21(1):1-17. doi: 10.1080/15476286.2024.2346688. Epub 2024 May 27. PMID: 38798162; PMCID: PMC11135835.;

[2] Meyer C, Garzia A, Mazzola M, Gerstberger S, Molina H, Tuschl T. The TIA1 RNA-Binding Protein Family Regulates EIF2AK2-Mediated Stress Response and Cell Cycle Progression. Mol Cell. 2018 Feb 15;69(4):622-635.e6. doi: 10.1016/j.molcel.2018.01.011. PMID: 29429924; PMCID: PMC5816707.
